# Unified characterization of the complete hierarchy of topological boundary states in a Floquet crystal

**DOI:** 10.1093/nsr/nwag170

**Published:** 2026-03-17

**Authors:** Yuxiang Guo, Zhoutao Lei, Jizhou Wu, Jiankun Zhu, Yao Qin, Linhu Li, Jingyun Fan

**Affiliations:** State Key Laboratory of Quantum Functional Materials and Department of Physics, Southern University of Science and Technology, Shenzhen 518055, China; Shenzhen Institute for Quantum Science and Engineering and Guangdong Provincial Key Laboratory of Quantum Science and Engineering, Southern University of Science and Technology, Shenzhen 518055, China; Guangdong Provincial Key Laboratory of Quantum Metrology and Sensing & School of Physics and Astronomy, Sun Yat-Sen University (Zhuhai Campus), Zhuhai 519082, China; Quantum Science Center of Guangdong-Hong Kong-Macao Greater Bay Area (Guangdong), Shenzhen 518045, China; State Key Laboratory of Quantum Functional Materials and Department of Physics, Southern University of Science and Technology, Shenzhen 518055, China; Shenzhen Institute for Quantum Science and Engineering and Guangdong Provincial Key Laboratory of Quantum Science and Engineering, Southern University of Science and Technology, Shenzhen 518055, China; State Key Laboratory of Quantum Functional Materials and Department of Physics, Southern University of Science and Technology, Shenzhen 518055, China; Guangdong Provincial Key Laboratory of Quantum Metrology and Sensing & School of Physics and Astronomy, Sun Yat-Sen University (Zhuhai Campus), Zhuhai 519082, China; Quantum Science Center of Guangdong-Hong Kong-Macao Greater Bay Area (Guangdong), Shenzhen 518045, China; State Key Laboratory of Quantum Functional Materials and Department of Physics, Southern University of Science and Technology, Shenzhen 518055, China; Shenzhen Institute for Quantum Science and Engineering and Guangdong Provincial Key Laboratory of Quantum Science and Engineering, Southern University of Science and Technology, Shenzhen 518055, China; Center for Advanced Light Source, Southern University of Science and Technology, Shenzhen 518055, China; Hefei National Laboratory, Hefei 230088, China

**Keywords:** topological phase, Floquet system, synthetic lattice, domain wall

## Abstract

Topological phases of matter are defined by bulk invariants that dictate the existence of robust boundary states. While conventional bulk–boundary correspondence links a given bulk invariant to a specific type of boundary mode, many systems may host multiple orders of topology simultaneously, including chiral edge states, weak edge states, and higher-order hinge and corner states. A unified framework for predicting and classifying all such boundary phenomena has remained elusive. Here, we introduce a compact topological triplet—three complementary one-dimensional winding numbers defined in distinct momentum subspaces—that fully determines the presence and type of boundary states in two-dimensional Floquet crystals. We experimentally implement this concept in a time-synthetic photonic lattice, demonstrating its predictive power. This unified approach integrates strong, weak, and higher-order topology into a single framework, providing critical insight for studying topological matter and enabling systematic control of complex topological phases across a broad range of physical platforms.

## Teaser

A unified framework captures strong, weak, and higher-order topological boundary states, demonstrated in a photonic Floquet lattice.

## INTRODUCTION

Topological quantum phases establish a profound connection between quantized observables and the global properties of band structures [1,2]. Higher-order topological phases (HOTPs) extend the conventional bulk–boundary correspondence by hosting gapless modes on boundaries of codimension greater than one, such as corner or hinge states, in addition to conventional edge states [3–8]. Over the past decade, a variety of theoretical methods were developed to predict and characterize HOTPs, including nested Wilson loop [3,4,9,10], nested band inversion surfaces [7,8], two-dimensional (2D) Zak phase [11,12], and diagonal winding numbers [13–16]. Experimentally, HOTPs were implemented across diverse platforms, including quantum materials [17–23], acoustic metamaterials [24–36], electric circuits [13,37], photonic lattices [38–41], and Floquet-engineered crystals [42,43]. In parallel, weak topological phases—characterized by lower-dimensional invariants embedded in higher-dimensional systems—have revealed novel boundary transport phenomena sensitive to lattice geometry and symmetry [44–46].

Despite these advances, existing characterization schemes are predominantly order-specific: distinct topological invariants are defined for different boundary orders, often requiring separate treatments. This fragmented approach complicates the characterization of systems where multiple orders of topology coexist or interact. A unified description capable of predicting all boundary phenomena from a single set of bulk invariants would significantly streamline both theory and experiments.

In this work, we address this challenge by introducing topological triplet invariants—three complementary one-dimensional winding numbers defined in distinct momentum subspaces—that fully predicts the presence and type of boundary states across multiple orders within a single two-dimensional Floquet crystal, from first-order chiral edge modes to weak topological states [44–46] and second-order corner-localized modes [3–8]. We implement this framework experimentally in a time-synthetic photonic lattice with complete parameter tunability and spatiotemporal resolution, enabling direct observation of strong, weak, and higher-order topological states. Our results establish an experimentally validated classification of chiral, weak, and higher-order boundary states within a single system, providing a broadly applicable strategy for designing and controlling complex topological phases in photonics, ultracold atoms, acoustics, and beyond.

## UNIFIED CHARACTERIZATION OF THE COMPLETE HIERARCHY OF TOPOLOGICAL BOUNDARY STATES

We consider a 2D Floquet lattice whose dynamics is governed by the Floquet operator:


(1) \begin{eqnarray*}
U=U_{\mathrm{x}}U_{\rm {y}},
\end{eqnarray*}

with $U_{\alpha } =T_\alpha R(\theta _\alpha )$ and $\alpha =\rm {x,\, y}$. Here, $R(\theta _\alpha )=e^{-i\frac{\sigma _{\rm {y}} \theta _\alpha }{2}}$ describes the coupling between, for example, the spin-up and spin-down states of a spin$-1/2$ particle or the horizontal ($\mathinner {|{H}\rangle }$ or $\mathinner {|{\uparrow }\rangle }$) and vertical polarization component ($\mathinner {|{V}\rangle }$ or $\mathinner {|{\downarrow }\rangle }$) of a laser pulse, and $T_{\alpha }=\sum _{\alpha }[|\alpha +1\rangle \langle \alpha |\otimes \mathinner {|{\uparrow }\rangle }\mathinner {\langle {\uparrow }|}+|\alpha -1\rangle \langle \alpha |\otimes \mathinner {|{\downarrow }\rangle }\mathinner {\langle {\downarrow }|}]$ implements polarization-dependent hopping along the $\alpha$ direction (see Note S1).

The corresponding Hamiltonian $H_{\rm F}=i\ln (U)$ and effective energy $E(\mathbf {k})$ are given by


(2) \begin{eqnarray*}
H_{\rm F}(\mathbf {k})&=&\frac{{E}(\mathbf {k})}{\sqrt{1-h^2_{0}}}(h_{\rm x}{\sigma }_{\rm x}+h_{\rm y}{\sigma }_{\rm y}+h_{\rm z}{\sigma }_{\rm z}), \\
E(\mathbf {k})&=&\arccos (h_{0}),
\end{eqnarray*}

with


\begin{eqnarray*}
h_{0}&=& -c_-s_{\rm x} s_{\rm y}+c_+ c_{\rm x} c_{\rm y}, \\
h_{x}&=& -s_-s_{\rm x} c_{\rm y}-s_+ c_{\rm x} s_{\rm y}, \\
h_{y}&=& -s_-s_{\rm x} s_{\rm y}+s_+c_{\rm x} c_{\rm y}, \\
h_{z}&=& c_-s_{\rm x} c_{\rm y}+ c_+ c_{\rm x} s_{\rm y},
\end{eqnarray*}

where $c_{\pm }=\cos (\frac{\theta _{\rm x}\pm \theta _{\rm y}}{2}), s_{\pm }=\sin (\frac{\theta _{\rm x}\pm \theta _{\rm y}}{2}), s_{\rm x}=\sin (k_{\rm x}), s_{\rm y}=\sin (k_{\rm y}), c_{\rm x}=\cos (k_{\rm x}), c_{\rm y}=\cos (k_{\rm y}),$ and ${\sigma }_\alpha$ with ${\alpha ={\rm x, y, z, {\rm and}\, 0}}$ denote Pauli matrices the $2\times 2$ identity matrix, respectively. $H_{\rm F}$ possesses a two-band quasienergy spectrum, exhibits distinct chiral symmetries $SH_{\rm F}S^{-1}=-H_{\rm F}$ within specific 1D momentum subspaces of the system. Specifically, the symmetry operator *S* takes the following forms: $S_0=\cos (\frac{\theta _{\rm x}-\theta _{\rm y}}{2})\sigma _{\rm x}+\sin (\frac{\theta _{\rm x}-\theta _{\rm y}}{2})\sigma _{\rm z}$ for subspaces $k_{\rm y}\in \lbrace 0,\pi \rbrace ({\rm or}\, k_{\rm x}\in \lbrace \pm \pi /2\rbrace )$, $S_{\pi /2}=\cos (\frac{\theta _{\rm x}+\theta _{\rm y}}{2})\sigma _{\rm x}+\sin (\frac{\theta _{\rm x}+\theta _{\rm y}}{2})\sigma _{\rm z}$ for subspaces $k_{\rm x}\in \lbrace 0,\pi \rbrace ({\rm or}\, k_{\rm y}\in \lbrace \pm \pi /2\rbrace )$, $S_+=\cos (\frac{\theta _{\rm x}}{2})\sigma _{\rm x}+\sin (\frac{\theta _{\rm x}}{2})\sigma _{\rm z}$ for subspaces $k_{-}\in \lbrace 0,\pi \rbrace$, and $S_-=\cos (\frac{\theta _{\rm x}}{2})\sigma _{\rm x}+\sin (\frac{\theta _{\rm x}}{2})\sigma _{\rm z}$ for subspaces $k_{+}\in \lbrace 0,\pi \rbrace$ with $k_{\pm }\equiv k_{\rm x}\pm k_{\rm y}$. A winding number can be defined for each of these chiral-symmetric subspaces, with three being independent (in Notes S1 and S2). Without loss of generality, the three representative ones for 1D subspaces with $k_{\rm y}=0$, $k_{\rm y}=\pi /2$, and $k_+=0$ are explicitly computed as [47–49]


(3) \begin{eqnarray*}
\nu _0 &=& \frac{-{\rm sign}\left[\sin \left(\frac{\theta _{\rm x}+\theta _{\rm y}}{2}\right)\right]}{2}, \\
\nu _{\pi /2}&=& \frac{-{\rm sign}\left[\sin \left(\frac{\theta _{\rm x}-\theta _{\rm y}}{2}\right)\right]}{2}, \\
\nu _{+}&=& {\rm sign}\left(\sin \frac{\theta _{\rm x}}{2}\right) \frac{{\rm sign}[\cos (\theta _{\rm x})-\cos (\theta _{\rm y})]\! -\! 1}{4} \\
&&+\,\theta _{\rm x}\leftrightarrow \theta _{\rm y},
\end{eqnarray*}

which yield quantized values $\nu _0,\, \nu _{\pi /2},\, \nu _{+}=\pm \frac{1}{2}$. Consequently, the 2D system supports eight topologically distinguished phases characterized by the triplet ($\nu _0,\, \nu _{\pi /2},\, \nu _{+}$).

**Figure 1.**
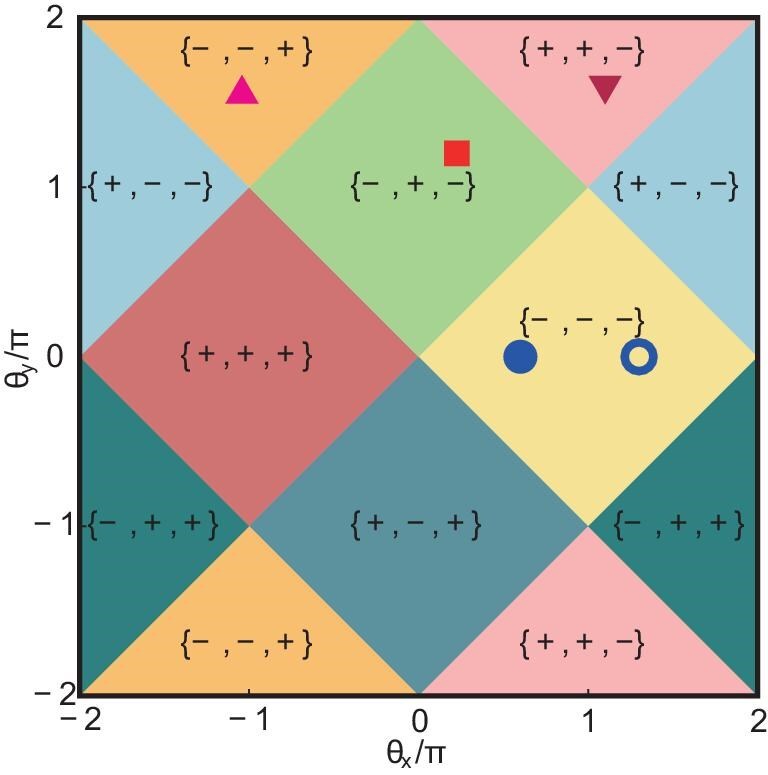
Phase diagram of the 2D lattice. The phase diagram comprises eight distinct topological phases characterized by triplet $(\nu _{0},\nu _{\pi /2},\nu _{+})$, with phase boundaries defined by $\theta _{\rm {x}}\pm \theta _{\rm {y}}=n\pi$, where $n\in \mathbb {Z}$. The symbols 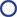, 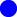, 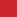, 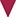, and 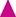 denote the parameter sets $(\theta _{\rm {x}},\theta _{\rm {y}})=(1.3\pi ,0)$, $(0.6\pi ,0)$, $(0.3\pi ,1.2\pi )$, $(1.1\pi ,1.6\pi )$, and $(-1.042\pi ,1.6\pi )$, respectively, and corresponding topological phases investigated in this study.

Figure 1 displays the phase diagram in the ($\theta _{\rm x},\, \theta _{\rm y}$) parameter space, with phase boundaries determined by the bandgap closings of the Hamiltonian $H_{\rm F}$, which occurs at $(k_{\rm x}\in \lbrace 0,\pi \rbrace ,\, k_{\rm y}\in \lbrace 0,\pi \rbrace )$ when $\theta _{\rm x}+\theta _{\rm y}=n\pi$, and $(k_{\rm x}\in \lbrace \pm \pi /2\rbrace ,k_{\rm y}\in \lbrace \pm \pi /2\rbrace )$ when $\theta _{\rm x}-\theta _{\rm y}=n\pi$ with $n\in \mathbb {Z}$. This rich structure of topological phases enables the realization of an entire set of topological boundary states, as shown in Table 1 and illustrated below.

**Table 1.** Unified characterization of the complete hierarchy of topological boundary states by a triplet of topological invariants.

Topological invariants	$\Box$	$\Diamond$
$\nu _{0}\cdot \nu _{\pi /2}$	$\overrightarrow{\!\!\!\Box }$ , strong	$\overrightarrow{\!\!\Diamond }$ , strong
$\left(\nu _{0}, \nu _{\pi /2}\right)$	$\Box$ , weak	$\diagdown \diagdown$ or $\diagup \diagup$, weak
$\nu _{+}\, \left({{\rm with}}\, \nu _{0},\nu _{\pi /2} { {\rm fixed}}\right)$	$:\, :$ , $2{{\rm nd}}$-order	$\Diamond$ , weak

The symbols $\Box$ and $\Diamond$ denote square and diamond domain wall geometries, ${\rightarrow_{\!\!\!\!\!\!\!\bullet }}$ indicates the direction of chirality, and $\diagdown \diagdown$ or $\diagup \diagup$ indicate the alignment of boundary states along the domain wall.

**Figure 2.**
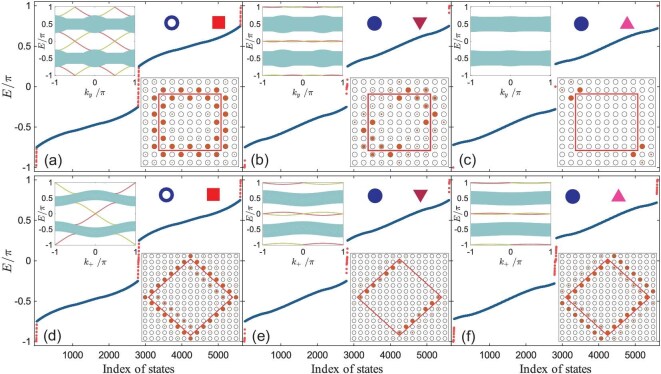
Spectral analysis of the 2D lattice. Used in pairs, the first and second symbols (same as Fig. 1), respectively, denote the topological properties of the inner and outer regions, which are separated by either a square domain wall (a–c) or a diamond domain wall (d–f). Panels (a–c) and (d–f) show the energy spectra. Insets depict the dispersion relations and representative topological eigen boundary states. The full 2D lattice spans $53\times 53$ with the square domain boundary spanning $7\times 7$ sites, and diamond domain boundary reaching 13 sites along both diagonal axes.

Consider a rectangular boundary geometry. When the inner and outer regions possess topological triplets, for example, $(-,-,\pm )$ and $(-,+,\pm )$ (omitting the factor of $\frac{1}{2}$ for simplicity), the resulting difference in Rudner–Lindner–Berg–Levin invariants [50–54], defined as $W[U]=4\nu _0\nu _{\pi /2}$, gives rise to topological band inversion at exactly one momentum point, $k_{\rm y} \in \lbrace 0, \pi /2\rbrace$, a hallmark of an anomalous Floquet insulator. In this case, the gapless edge states in the spectrum and their spatially extended nature along the entire domain wall, as shown in Fig. 2a, indicate the existence of chiral boundary states that characterize a strong first-order topology.

In contrast, when the inner and outer regions satisfy $\nu ^{\rm in}_{0}\nu ^{\rm out}_{0}=-1$ and $\nu ^{\rm in}_{\pi /2}\nu ^{\rm out}_{\pi /2}=-1$, that is, simultaneous mismatch in both $\nu _0$ and $\nu _{\pi /2}$, band inversion occurs at both $k_{\rm y}=0$ and $k_{\rm y}=\pi /2$. This configuration supports boundary states gapped from the bulk bands (Fig. 2b). Although these modes delocalize along the domain wall, their dispersion crosses zero energy multiple times, yielding nearly zero average group velocity and negligible net transport, characteristic of weak topological phases [44].

For regions where $\nu ^{\rm in}_{0} \nu ^{\rm out}_{0} = 1$, $\nu ^{\rm in}_{\pi /2} \nu ^{\rm out}_{\pi /2} = 1$, and $\nu ^{\rm in}_{+} \nu ^{\rm out}_{+} = -1$, that is, $\nu _0$ and $\nu _{\pi /2}$ remain unchanged while $\nu _+$ flips across the boundary, the resulting characteristic singular energy spectrum and the pronounced localization of boundary states at the intersection of orthogonal domain walls, as shown in Fig. 2c, signify the emergence of second-order topological corner states [13,16].

Figure 2d–f present the theoretical boundary states under the same topological conditions, with the square domain wall geometry replaced by a diamond-shaped configuration. In the first scenario, the chiral boundary states persist irrespective of the boundary geometry (Fig. 2d). In the second scenario, while the appearance of weak boundary states are independent of the value of $\nu _{+}$ in the rectangular domain wall, the value of $\nu _{+}$ governs the emergence and spatial orientation of non-chiral boundary states along the diamond domain wall. Specifically, when $\nu _{+}$ differs between the inner and outer regions, the unchiral boundary states localize along the domain walls parallel to the $x+y$ direction (Fig. 2e). Conversely, when $\nu _+$ is the same in both regions, these states appear along the $x-y$ direction (see Note S6A). Notably, in the third scenario, the second-order corner states are absent. This absence arises from the system’s capacity to support nontrivial topology along the $x+y$ and $x-y$ directions, even when the *x* and *y* directions are topologically trivial. In such cases, a weak topological boundary state emerges instead, as shown in Fig. 2f. In contrast, the inverse scenario, where both *x* and *y* directions are nontrivial but the $x+y$ and $x-y$ directions are trivial, is topologically forbidden. This study demonstrates that the existence and spatial localization of weak topological and second-order boundary states are critically determined by the system’s boundary configuration, that is, exhibiting geometry-dependence (see detailed theoretical analysis in Note S3 and the additional experiments in Note S6B).

Although static spatial profiles may appear similar for certain chiral and non-chiral boundary states, their distinct dynamical evolution enables unambiguous experimental discrimination of all boundary states in our system.

## EXPERIMENTAL OBSERVATION OF DYNAMICS

**Figure 3.**
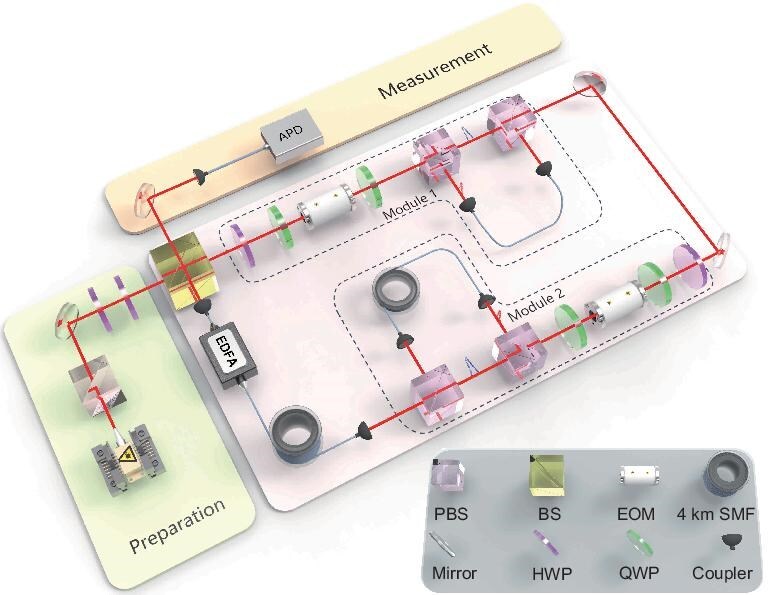
Experimental schematic. A laser pulse (1560 nm, 0.5 ns) is injected into an optical loop via a beam splitter (BS). Inside the loop, the pulse sequentially traverses two modules that implement the operations $U_{\rm x}$ and $U_{\rm y}$, respectively. Each module consists of a set of half-wave (HWP) and quarter-wave plates together with an electro-optic modulator (EOM) for realizing the rotation $R(\theta _\alpha )$, and an unbalanced interferometer for implementing the shift $T_\alpha$. The interferometer comprises two polarization beam splitters (PBSs) and a segment of single-mode fiber (SMF) with lengths of 5.60 m in the first module and 151.10 m in the second module, respectively. A 4 km SMF is employed to allow sufficient temporal separation between time steps. Fifteen percent intensity of the laser pulse is tapped out via the BS after each circulation to perform spatiotemporal measurements by an APD. An erbium-doped fiber amplifier (EDFA) is incorporated to compensate for optical losses. The laser pulse, EOM, and detection are synchronized (see Note S4). The experiment is repeated at a rate of 400 Hz.

We experimentally realize the proposed 2D Floquet crystal hosting the complete hierarchy of boundary states in a photonic synthetic lattice. As illustrated in Fig. 3, this is achieved through controlled circulation of laser pulses in a dynamically modulated optical loop network, enabling precise observation of all topological boundary regimes. In each iteration, the pulse sequentially traverses two modules. Each contains a waveplate assembly implementing the rotation operation $R(\theta _\alpha )$ and an unbalanced Mach–Zehnder interferometer performing the hopping operation $T_\alpha$, where the path-length difference between the two arms sets the time-bin seperation $\tau _\alpha$. With $\tau _{\rm x}=$ 13.99 ns and $\tau _{\rm y}=$ 377.76 ns in the first and second modules, respectively, we create a 2D synthetic lattice ($x,\, y$). In the following experiments, the laser pulses are initially confined to a single lattice site located on the domain wall, prepared in the state $\mathinner {|{\psi _0}\rangle }$. The system then evolves discretely under repeated application of the Floquet operator, such that the state after *n* loop circulations is given by $\mathinner {|{\psi _n}\rangle } = U^n \mathinner {|{\psi _0}\rangle }$. To track the evolution of boundary states, we perform time-resolved measurements by weakly tapping the light signal after each cycle and detect the outcoupled intensity using a high-speed avalanche photodiode (APD). This enables reconstruction of the full spatiotemporal intensity distribution, $p(x, y, n) = |\langle x, y, \uparrow | \psi _n \rangle |^2 + |\langle x, y, \downarrow | \psi _n \rangle |^2$. The results are shown in Fig. 2. In each subdiagram, the top row presents experimental measurements and the bottom row shows numerical simulations. The experimental data are recorded for up to 26 time steps, after which the signal reaches the noise floor.

**Figure 4.**
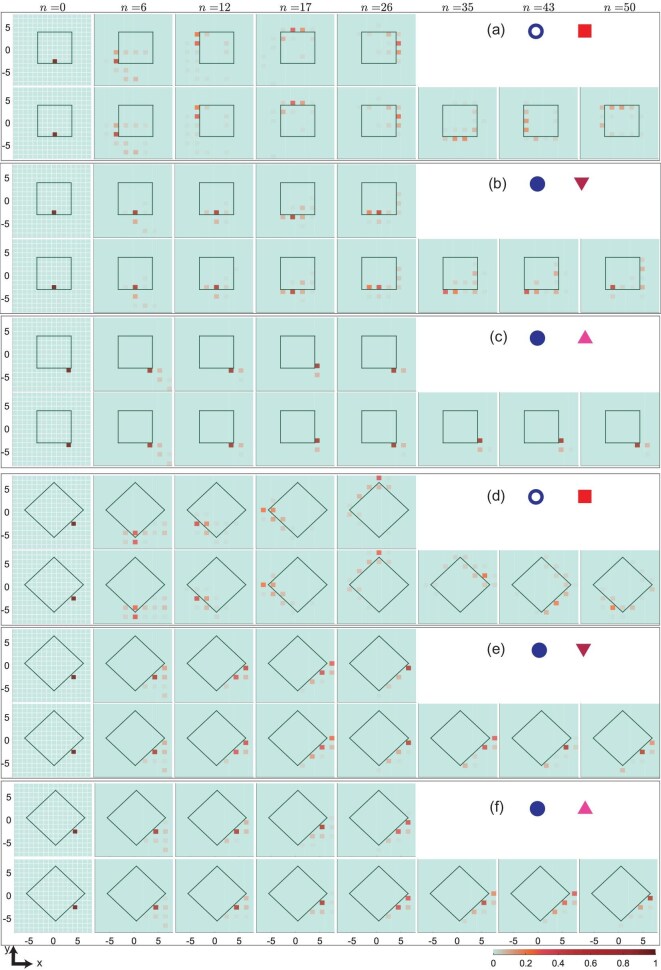
Spatiotemporally resolved measurements of the laser pulse intensity distribution across the 2D lattice for (a–c) rectangular and (d–f) diamond domain wall configurations at time steps $n=0, 6, 12, 17, 26, 35, 43, {\rm and}\, 50$. The upper (lower) panel is the experimental (theoretical) dynamics in panels (a–f). The topological triplet invariants for the inner and outer regions of the domain wall are: (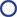, 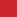) [$(-, -, -)$, $(-, +, -)$] in panels (a and d), (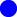, 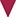) [$(-, -, -)$, $(+, +, -)$] in panels (b and e), (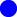, 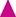) [$(-, -, -)$, $(-, -, +)$] in panels (c and f), respectively. The color bar indicates the intensity level of the laser pulse at each lattice site.

First, we set $\boldsymbol {\theta }^{{\rm in}}=(\theta _{\rm x}, \theta _{\rm y})=(1.3\pi , 0)$ and $\boldsymbol {\theta }^{{\rm out}}=(0.3\pi , 1.2\pi )$, creating inner and outer regions with topological triplets $(\nu _0, \nu _{\pi /2}, \nu _{\pi })=(-,-,-)$ and $(-,+,-)$, respectively. This induces a mismatch in the Rudner–Lindner–Berg–Levin invariant across the boundary. Launching the pulse from the center of the bottom boundary, we observe unidirectional propagation along both square and diamond domain walls, as shown in Fig. 4a and d, unambiguously demonstrating the presence of chiral edge states.

In the second experiment, with $\boldsymbol {\theta }^{{\rm in}}= (0.6\pi ,\, 0)$ and $\boldsymbol {\theta }^{{\rm out}}=(1.1\pi,\, 1.6\pi)$, the inner and outer regions are set to triplets $(-,-,-)$ and $(+,+,-)$, respectively, satisfying $\nu ^{\mathrm{in}}_{0}\nu ^{\mathrm{out}}_{0}=-1$ and $\nu ^{\mathrm{in}}_{\pi /2} \nu ^{\mathrm{out}}_{\pi /2}=-1$. In this configuration, the laser pulse, initially launched at the center of the bottom boundary, gradually delocalizes and accumulates small displacement over time, as shown in Fig. 4b and e, indicating the presence of weak topological boundary states.

In the third experiment, with $\boldsymbol {\theta }^{{\rm in}}= (0.6\pi ,\, 0)$ and $\boldsymbol {\theta }^{{\rm out}}=(-1.042\pi ,\, 1.6\pi )$, we realize an inner region with triplet ($-,-,-$) and an outer region with ($-,-,+$), satisfying $\nu ^{\mathrm{in}}_{0} \nu ^{\mathrm{out}}_{0} = 1$, $\nu ^{\mathrm{in}}_{\pi /2} \nu ^{\mathrm{out}}_{\pi /2} = 1$, and $\nu ^{\mathrm{in}}_{+} \nu ^{\mathrm{out}}_{+} = -1$. Spatiotemporal measurements reveal that the pulse intensity predominantly oscillates between two lattice sites near the corner, indicating strong localization (Fig. 4c). In contrast, in the diamond domain-wall geometry, the pulse exhibits delocalization and slow displacement along the boundary (Fig. 4f), consistent with non-chiral boundary states.

Notably, the initial state is set to $R^{-1}(\frac{\theta _{\rm x}^{{\rm in}}-\theta _{\rm y}^{{\rm in}}-\pi }{2})\mathinner {|{x=0,y=-3,\, \uparrow }\rangle }$ in Fig. 4a and b, $R^{-1}(\frac{\theta _{\rm x}^{{\rm out}}-\pi }{2})\mathinner {|{x=3,y=-4,\, \uparrow }\rangle }$ in Fig. 4c, and $R^{-1}(\frac{\theta _{\rm x}^{{\rm out}}+2\pi }{2})\mathinner {|{x=4,y=-3,\, \uparrow }\rangle }$ in Fig. 4d–f, to maximize the overlap with the corresponding eigenstate in each of the scenario. Numerical simulations consistently reproduce the experimental observations under identical conditions, validating both our theoretical framework and experimental implementation. This agreement further enables numerical exploration over extended evolution times beyond the experimental window, thereby more clearly revealing the dynamical features (Fig. 4).

To further characterize the dynamics, we analyze both the center-of-mass displacement and the inverse participation ratio (IPR) as functions of step number *n*. The center-of-mass is computed as $\langle {-6}\tilde{\psi}_n | x_{c} | {-6}\tilde{\psi }_n \rangle /\mathinner {\langle {{-6}\tilde{\psi }_{n}|{-6}\tilde{\psi }_{n}}\rangle }$, representing the expectation value of the position operator $x_{c}$ along the boundary at step *n*, where $\mathinner {|{{-6}\tilde{\psi }}\rangle }$ is the state projected onto sites near domain walls (see Note S5). For domain wall transport analysis, we project this position onto the domain wall axis to obtain the propagation distance ($d_{\rm c}$). The IPR is defined as ${\rm IPR}=\sum _{(x,y)}\left(|\langle x,y,\uparrow |\psi _n\rangle |^2 + |\langle x,y,\downarrow |\psi _n\rangle |^2\right)^2$, where the summation runs over all lattice sites $(x,\, y)$. In the thermodynamic limit, the IPR approaches zero for fully extended states and unity for perfectly localized states.

**Figure 5.**
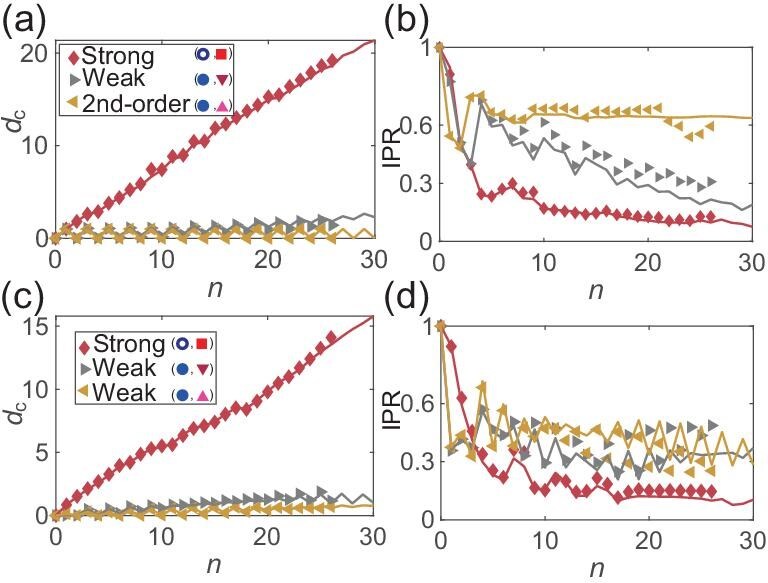
The center-of-mass ($d_{\rm c}$) and IPR are plotted as functions of the time step *n*. Panels (a and b) and (c and d) correspond to the square and diamond domain wall configurations, respectively. Symbols represent experimental data, while smooth lines denote numerical simulations.

As shown in Fig. 5a, the time evolution of center-of-mass reveals three distinct dynamical regimes for the square domain wall configuration: linear growth with a large slope (diamond markers), linear growth with a small but finite slope (right triangle), and bounded oscillations without growth (left triangle), which correspond to the three classes of boundary states: strong, weak, and second-order, respectively. We emphasize that, although the slope for the weak boundary states is small, its finite value clearly distinguishes them from second-order boundary states, which exhibit zero growth. Furthermore, Fig. 5b shows that the IPR provides complementary information. While the IPR stabilizes around $\sim 0.6$ for the second-order boundary state, it continuously decreases to values well below 0.6 for both strong and weak boundary states. Therefore, whereas the time evolution of the center-of-mass distinguishes strong boundary states from weak and second-order ones, the IPR differentiates second-order boundary states from strong and weak ones. Similarly, as shown in Fig. 5c, the time evolution of the center-of-mass provides a reliable measure for distinguishing chiral boundary states—characterized by linear growth with a large slope—from weak boundary states, which exhibit linear growth with a small but finite slope in the diamond domain wall configuration. The different slopes of the two types of weak boundary states originate from their distinct dispersion relations (Fig. 2e and f). We further observe that the IPR values for both strong and weak boundary states decrease on nearly the same time scale, and therefore do not provide a reliable measure for distinguishing between them within the experimental time window, as shown in Fig. 5d. Across all measurements, experimental data show excellent agreement with theoretical predictions (solid lines), validating the triplet-invariant framework for capturing and unifying multiple orders of boundary topology in a single system.

## CONCLUSIONS

In summary, we establish a unified framework for controlling the complete hierarchy of boundary states in a two-dimensional Floquet crystal. Using a minimal set of topological triplet invariants—each corresponding to a winding number in a distinct 1D momentum subspace, our formalism provides a complete characterization not only for the strong, weak, and 2nd-order topological phases in our system, but also their divergent geometry-dependent behaviors at domain walls aligned with the subspaces. This work advances our understanding and realization of higher order topological phases while providing a versatile experimental platform for investigating exotic boundary phenomena in synthetic quantum systems and driven topological matter. Moreover, our unified characterization of the complete hierarchy of topological boundary states in synthetic time dimensions can be readily extended to other experimental platforms in which the dynamical evolution can be directly monitored, such as cold atom [55], trap ions [56], and electronic circuit system [57].

## METHODS

### Definition of triplet topological invariant

Based on the symmetry analysis of our system, which is detailed in Note S2, we define the triplet topological invariants. Take the $k_{\rm y}=0$ subspace holding chiral symmetry $S_0U(k_{\rm x},0)S_0=U^{-1}(k_{\rm x},0)$ with $S_0=\cos (\frac{\theta _{\rm x}-\theta _{\rm y}}{2})\sigma _{\rm x}+\sin (\frac{\theta _{\rm x}-\theta _{\rm y}}{2})\sigma _{\rm z}$ as an example to demonstrate how to define the winding numbers in these chiral symmetric subspaces. With $R(\frac{\theta _{\rm x}-\theta _{\rm y}-\pi }{2})S_0R^{-1}(\frac{\theta _{\rm x}-\theta _{\rm y}-\pi }{2})=\sigma _{\rm z}$, the effective Hamiltonian can be written in the off-diagonal form as


(4) \begin{eqnarray*}
&& \!\!R\left(\frac{\theta _{\rm x}-\theta _{\rm y}-\pi }{2}\right)H_{{\rm F}}(k_{\rm x},0)R^{-1}\left(\frac{\theta _{\rm x}-\theta _{\rm y}-\pi }{2}\right) \\
&&=\frac{\arccos (h_{0})}{\sqrt{1-h^2_{0}}}
\left({\begin{array}{l c}
0&\quad d_0(k_{\rm x})\\
d^{*}_0(k_{\rm x})&\quad 0 \end{array}}\right),
\end{eqnarray*}

where $H_{{\rm F}}(k_{\rm x},0)=i{\rm ln}[U(k_{\rm x},0)]$ and $d_0(k_{\rm x})=-\sin (k_{\rm x})-i\sin (\frac{\theta _{\rm x}+\theta _{\rm y}}{2})\cos (k_{\rm x})$. Then, the corresponding 1D winding number is


(5) \begin{eqnarray*}
\tilde{\,\,\nu }_0 &=& \frac{1}{2\pi i}\int _{-\pi }^{\pi }dk_{\rm x}\frac{d}{dk_{\rm x}}\ln \left[\frac{\arccos (h_{0})}{\sqrt{1-h^2_{0}}}d_0(k_{\rm x})\right] \\
&=& -{\rm sign}\left[\sin \left(\frac{\theta _{\rm x}+\theta _{\rm y}}{2}\right)\right],
\end{eqnarray*}

with $h_{0}=\cos (\frac{\theta _{\rm x}+\theta _{\rm y}}{2})\cos (k_{\rm x})$. This topological invariant actually describes the net number of edge states in the 0 gap and $\pi$ gap, considering their polarization (the expectation value of the chiral symmetry operator, and the edge states are eigenstates of this operator) are either $+1$ or $-1$. To define the winding number for the 0 gap solely, we introduce two auxiliary operators:


(6) \begin{eqnarray*}
F_0 &=& R(\theta _{\rm y})T_{\rm x}^{(-)}R\left(\frac{\theta _{\rm x}-\theta _{\rm y}}{2}\right), \\
T_{\rm x}^{(-)} &=& \Big({\begin{array}{cc} 1 & 0 \\
0 & e^{ik_{\rm x}} \end{array}}\Big), \\
G_0 &=& R^{-1}\left(\frac{\theta _{\rm x}-\theta _{\rm y}}{2}\right)T_{\rm x}^{(+)}R(\theta _{\rm x}), \\
T_{\rm x}^{(+)} &=&
\left({\begin{array}{cc}
e^{-ik_{\rm x}} &\quad 0 \\
0 &\quad 1 \end{array}}\right),
\end{eqnarray*}

which satisfy the property $S_0F_0S_0G_0 = \sigma _0$ with $\sigma _0$ being the $2\times 2$ identity matrix. Obviously, $U(k_{\rm x},0) = F_0G_0$, and another unitary operator can be defined as $U^{\prime }(k_{\rm x},0)=G_0F_0$, which also satisfies the chiral symmetry $S_0U^{\prime }(k_{\rm x},0)S_0 = U^{\prime -1}(k_{\rm x},0)$. Similarly, we can define the winding number for its effective Hamiltonian $H^{\prime }_{\rm F}(k_{\rm x},0)= i{\rm ln}[U^{\prime }(k_{\rm x},0)]$ as


(7) \begin{eqnarray*}
\tilde{\,\,v}^{\prime }_0 &=& \frac{1}{2\pi i}\int _{-\pi }^{\pi }dk_{\rm x} \frac{d}{dk_{\rm x}}\ln \left[\frac{\arccos (h_0)}{\sqrt{1-h_0^2}}d^{\prime }_0(k_{\rm x})\right]\\
&=& 0,
\end{eqnarray*}

with $d^{\prime }_0(k_{\rm x})=-\cos (\frac{\theta _{\rm x}+\theta _{\rm y}}{2})\sin k_{\rm x}-i\sin (\frac{\theta _{\rm x}+\theta _{\rm y}}{2})$. However, since $U(k_{\rm x},0)$ and $U^{\prime }(k_{\rm x},0)$ are linked through a unitary transformation $U^{\prime }(k_{\rm x},0) = G_0U(k_{\rm x},0)G_0^{-1}$, they have the same energy spectrum and the same number of boundary states at each gap. Furthermore, the polarization is the same for the zero-energy boundary states of these two operators, while the polarization is opposite for the $\pi$-energy boundary states [48]. We can thus obtain the winding number for the 0 gap of $U(k_{\rm x},0)$:


(8) \begin{eqnarray*}
\nu _0=\frac{\tilde{\,\,v}_0 + \tilde{\,\,v}^{\prime }_0}{2}=\frac{-\mathrm{sign}\left[\sin \left(\frac{\theta _{\rm x}+\theta _{\rm y}}{2}\right)\right]}{2},
\end{eqnarray*}

which indicates the nontrivial band inversion in the subspace $k_{\rm y}=0$ within 0 gap.

Using this approach, we can also obtain the winding number for the subspace $k_{\rm y}=\pi /2$ in the 0 gap, denoted as $\nu _{\pi /2}$, and the winding number for the subspace $k_-=0$ in the 0 gap, denoted as $\nu _+$, as provided in Eq. (3). Actually, there are more than three winding numbers in chiral symmetric subspaces within the 0 or $\pi$ gap of our 2D Floquet quantum system. However, only the three winding numbers $\lbrace \nu _0,\nu _{\pi /2},\nu _+\rbrace$ are independent and can be used to characterize all chiral symmetric 1D subspaces. The detailed theoretical analysis is provided in Note S2.

### Experimental setup

In order to experimentally realize the 2D Floquet crystal, we use the light pulse’s polarization to represent the Floquet operator’s intrinsic spin and time-bins in the time dimension to encode the Floquet operator’s states in the spatial dimension. The input light pulses are generated by a distributed feedback laser (with a central wavelength of 1560 nm and a repetition rate of 400 Hz) and later truncated by two intensity modulators with 30 dB extinction ratio in sequence, resulting in the pulses with 0.5 ns pulse width. Two HWPs rotate the pulses’ polarization to initialize the internal state of the operator *U*. An HWP and EOM sandwiched between two quarter-wave plates implement the $R(\theta )$ operator of *x* (*y*) dimension. Two PBSs combined with a 5.60 m (151.10 m) long single-mode fiber perform the $T_x$ ($T_y$) operator. An EDFA is used to compensate for the optical losses. The output pulse after circulation in each Floquet step is detected by an APD. The signals from the APD are read by a high-speed oscilloscope (8 GHz bandwidth and 40 GSam/s sampling rate).

## Supplementary Material

nwag170_Supplemental_File
