# Supplementary material for: Unified characterization of the complete hierarchy of topological boundary states in a Floquet crystal
Source: Natl Sci Rev. 2026 Mar 17;13(12):nwag170. doi: 10.1093/nsr/nwag170 (PMC13256033; doi:10.1093/nsr/nwag170)
Supplement: nwag170_Supplemental_File [file nwag170_supplemental_file.pdf]

Supplementary Note for "Unified Characterization of the Complete Hierarchy of Topological Boundary States in a Floquet Crystal"

Supplementary Note 1. THE EFFECTIVE HAMILTONIAN AND 1D CHIRAL SYMMETRIC SUBSPACES

The Floquet operator U in Eq. (1) of the main text is given by

$$\begin{aligned} U &= T_y R(\theta_y) T_x R(\theta_x) \\ R(\theta_x) &= e^{-i\frac{\sigma_y\theta_x}{2}} \\ R(\theta_y) &= e^{-i\frac{\sigma_y\theta_y}{2}} \\ T_x &= \sum_x [|x+1\rangle\langle x| \otimes |\uparrow\rangle\langle\uparrow| + |x-1\rangle\langle x| \otimes |\downarrow\rangle\langle\downarrow|] \\ T_y &= \sum_y [|y+1\rangle\langle y| \otimes |\uparrow\rangle\langle\uparrow| + |y-1\rangle\langle y| \otimes |\downarrow\rangle\langle\downarrow|]. \end{aligned} \tag{S1}$$

Transforming to momentum space, the Floquet operator U can be expressed in terms of Pauli matrices as

$$\begin{aligned} U(\mathbf{k}) &= h_0\sigma_0 - i(h_x\sigma_x + h_y\sigma_y + h_z\sigma_z), \\ h_0 &= -\cos(\frac{\theta_x-\theta_y}{2})\sin(k_x)\sin(k_y) + \cos(\frac{\theta_x+\theta_y}{2})\cos(k_x)\cos(k_y), \\ h_x &= -\sin(\frac{\theta_x-\theta_y}{2})\sin(k_x)\cos(k_y) - \sin(\frac{\theta_x+\theta_y}{2})\cos(k_x)\sin(k_y), \\ h_y &= -\sin(\frac{\theta_x-\theta_y}{2})\sin(k_x)\sin(k_y) + \sin(\frac{\theta_x+\theta_y}{2})\cos(k_x)\cos(k_y), \\ h_z &= \cos(\frac{\theta_x-\theta_y}{2})\sin(k_x)\cos(k_y) + \cos(\frac{\theta_x+\theta_y}{2})\cos(k_x)\sin(k_y), \end{aligned} \tag{S2}$$

where $\sigma_{\alpha=x,y,z}$ denote Pauli matrices and σ_0 denotes the 2×2 identity matrix. The effective energy $E(\mathbf{k})$ and Hamiltonian $H_{\mathrm{F}}(\mathbf{k}) = i\ln(U(\mathbf{k}))$ can be expressed as:

$$\begin{aligned} E(\mathbf{k}) &= \arccos(h_0), \\ H_{\mathrm{F}}(\mathbf{k}) &= \frac{E(\mathbf{k})}{\sqrt{1-h_0^2}}(h_x\sigma_x + h_y\sigma_y + h_z\sigma_z). \end{aligned} \tag{S3}$$

This system possesses inversion symmetry, which is mathematically expressed as

$$\sigma_y U(\mathbf{k})\sigma_y = U(-\mathbf{k}), \tag{S4}$$

$$\sigma_y H_{\mathrm{F}}(\mathbf{k})\sigma_y = H_{\mathrm{F}}(-\mathbf{k}), \tag{S5}$$

a particle-hole symmetry

$$U(\mathbf{k}) = U^*(-\mathbf{k}), \tag{S6}$$

$$H_{\mathrm{F}}(\mathbf{k}) = -H_{\mathrm{F}}^*(-\mathbf{k}), \tag{S7}$$

a time-reversal symmetry

$$\mathcal{T}U(\mathbf{k})\mathcal{T}^{-1} = U^{-1}(-\mathbf{k}), \tag{S8}$$

with

$$\mathcal{T} = [\cos(\frac{\theta_x+\theta_y}{2})\sigma_x + \sin(\frac{\theta_x+\theta_y}{2})\sigma_z]\mathcal{K}. \tag{S9}$$

Notably, time-reversal symmetry emerges when $\theta_y = n\pi$ ($n \in \mathcal{Z}$), with $\mathcal{K}$ denoting the complex conjugation operator. At these parameter values, the Chern number must vanish due to either time-reversal symmetry [1] or $\mathcal{PT}$ symmetry [2] (combination of

inversion and time-reversal symmetry). Because all of the other parameters with an energy gap can be adiabatically deformed to the case with $\theta_y = n\pi$, the Chern number for U must vanish, as established in Refs. [3, 4]. Instead, the topological properties of U in these works are characterized by the Rudner-Lindner-Berg-Levin winding number [5]. Furthermore, because chiral symmetry emerges in specific 1D subspaces, we introduce a triplet topological invariant composed of subspace winding numbers, which enriches the classification of topological phases.

In general, the chiral symmetry operator can be defined as $S = \vec{s}\cdot\vec{\sigma}$ with $\vec{\sigma} = (\sigma_x, \sigma_y, \sigma_z)$ and $\vec{s} = (s_x, s_y, s_z)$ being a **k**-independent unit vector. The effective Hamiltonian is represented by $H_{\mathrm{F}} = \frac{E(\mathbf{k})}{\sqrt{1-h_0^2}}\vec{h}\cdot\vec{\sigma}$ with $\vec{h} = (h_x, h_y, h_z)$ being a **k**-dependent vector. The chiral symmetry emerges when

$$(\vec{s}\cdot\vec{\sigma})(\vec{h}\cdot\vec{\sigma})(\vec{s}\cdot\vec{\sigma}) = -\vec{h}\cdot\vec{\sigma}. \tag{S10}$$

This leads to

$$\vec{s}\cdot\vec{h} = 0. \tag{S11}$$

The explicit form is given by

$$\sin(k_+)\cos\frac{\theta_y}{2}(\sin\frac{\theta_x}{2}s_x - \cos\frac{\theta_x}{2}s_z) - \sin(k_-)\sin\frac{\theta_y}{2}(\cos\frac{\theta_x}{2}s_x + \sin\frac{\theta_x}{2}s_z) = -[\cos(k_+)\sin\frac{\theta_x}{2}\cos\frac{\theta_y}{2} + \cos(k_-)\cos\frac{\theta_x}{2}\sin\frac{\theta_y}{2}]s_y, \tag{S12}$$

with $k_\pm \equiv k_x \pm k_y$. These equations are not generally satisfied in the full 2D **k** space, except for special parameter values (θ_x, θ_y) (such as $\theta_y = n\pi$). However, they can establish for some 1D subspaces.

Firstly, we note that there is no **k**-independent solution of $\vec{s}$ with $s_y \neq 0$, except for some special parameters, because the angle between vector $\vec{h}$ and $h_y = 0$ plane is always **k**-dependent even in 1D subspace of **k**. For the solution with $s_y = 0$, Eq. (S12) reduces to:

$$\sin(k_+)\cos\frac{\theta_y}{2}[\sin(\frac{\theta_x}{2} - \phi)] = \sin(k_-)\sin\frac{\theta_y}{2}[\cos(\frac{\theta_x}{2} - \phi)], \tag{S13}$$

with setting $\cos\phi = s_x$ and $\sin\phi = s_z$. We can obtain **k**-independent solution of ϕ in several subspaces with constant value of $\frac{\sin(k_+)}{\sin(k_-)}$:

1. $k_y = 0, \pi$ and $k_x = \pm\frac{\pi}{2}$ subspaces, where $\sin(k_+) = \sin(k_-)$, the solution will be $\phi = \frac{\theta_x - \theta_y}{2}$, indicating the chiral symmetric operator will be $S_0 = \cos(\frac{\theta_x-\theta_y}{2})\sigma_x + \sin(\frac{\theta_x-\theta_y}{2})\sigma_z$;

2. $k_y = \pm\frac{\pi}{2}$ and $k_x = 0, \pi$ subspaces, where $\sin(k_+) = -\sin(k_-)$, the solution will be $\phi = \frac{\theta_x+\theta_y}{2}$, indicating the chiral symmetric operator will be $S_{\pi/2} = \cos(\frac{\theta_x+\theta_y}{2})\sigma_x + \sin(\frac{\theta_x+\theta_y}{2})\sigma_z$;

3. $k_- = 0, \pi$ subspaces, where $\sin(k_-) = 0$, the solution will be $\phi = \frac{\theta_x}{2}$, indicating the chiral symmetric operator will be $S_+ = \cos(\frac{\theta_x}{2})\sigma_x + \sin(\frac{\theta_x}{2})\sigma_z$;

4. $k_+ = 0, \pi$ subspaces, where $\sin(k_+) = 0$, the solution will be $\phi = \frac{\theta_x-\pi}{2}$, indicating the chiral symmetric operator will be $S_- = \sin(\frac{\theta_x}{2})\sigma_x - \cos(\frac{\theta_x}{2})\sigma_z$;

all of these operators are summarized in Table S1. Based on the chiral symmetry, the winding number can be defined as discussed in Supplementary Note 2.

Supplementary Note 2. DEFINITION OF THE TRIPLET TOPOLOGICAL INVARIANTS

In Supplementary Note 1, we identified the chiral symmetric 1D subspaces. In this section, we define the winding numbers for these subspaces, respectively [6, 7].

Firstly, we investigate the case with $k_y = 0$: $U(k_x, 0) = R(\theta_y)T_xR(\theta_x)$, which holds a chiral symmetry:

$$S_0U(k_x, 0)S_0 = U^{-1}(k_x, 0) \tag{S14}$$

or equally

$$S_0H_{\mathrm{F}}(k_x, 0)S_0 = -H_{\mathrm{F}}(k_x, 0) \tag{S15}$$

TABLE S1. Other topological invariants expressed by triplet topological invariant $\{\nu_0^{(0)}, \nu_{\pi/2}^{(0)}, \nu_+^{(0)}\}$ with their chiral operator. Here $k_\pm \equiv k_x \pm k_y$, $W[U]$ is the Rudner-Lindner-Berg-Levin winding number for the Floquet operator U, and $W_{\boldsymbol{k}}^{\epsilon}$ is the 1D winding number in the ϵ-energy gap along the subspace $\boldsymbol{k}$.

Topological invariants	Values	Chiral symmetry operator (for 1D $W_{\boldsymbol{k}}^{\epsilon}$)
$W[U]$	$4\nu_0^{(0)}\nu_{\pi/2}^{(0)}$	\
$W_{k_y=0,\pi}^{\epsilon=0,\pi}$	$\nu_0^{(0)}$	$S_0 = \cos(\frac{\theta_x-\theta_y}{2})\sigma_x + \sin(\frac{\theta_x-\theta_y}{2})\sigma_z$
$W_{k_x=0,\pi}^{\epsilon=0,\pi}$	$\nu_0^{(0)}$	$S_{\pi/2} = \cos(\frac{\theta_x+\theta_y}{2})\sigma_x + \sin(\frac{\theta_x+\theta_y}{2})\sigma_z$
$W_{k_y=\pm\frac{\pi}{2}}^{\epsilon=0,\pi}$	$\nu_{\pi/2}^{(0)}$	$S_{\pi/2} = \cos(\frac{\theta_x+\theta_y}{2})\sigma_x + \sin(\frac{\theta_x+\theta_y}{2})\sigma_z$
$W_{k_x=\pm\frac{\pi}{2}}^{\epsilon=0,\pi}$	$\nu_{\pi/2}^{(0)}$	$S_0 = \cos(\frac{\theta_x-\theta_y}{2})\sigma_x + \sin(\frac{\theta_x-\theta_y}{2})\sigma_z$
$W_{k_-=0}^{\epsilon=0}$ and $W_{k_-=\pi}^{\epsilon=\pi}$	$\nu_+^{(0)}$	$S_+ = \cos(\frac{\theta_x}{2})\sigma_x + \sin(\frac{\theta_x}{2})\sigma_z$
$W_{k_-=0}^{\epsilon=\pi}$ and $W_{k_-=\pi}^{\epsilon=0}$	$4\nu_0^{(0)}\nu_{\pi/2}^{(0)}\nu_+^{(0)}$	$S_+ = \cos(\frac{\theta_x}{2})\sigma_x + \sin(\frac{\theta_x}{2})\sigma_z$
$W_{k_+=0}^{\epsilon=0}$ and $W_{k_+=\pi}^{\epsilon=\pi}$	$2\nu_{\pi/2}^{(0)}\nu_+^{(0)}$	$S_- = \sin(\frac{\theta_x}{2})\sigma_x - \cos(\frac{\theta_x}{2})\sigma_z$
$W_{k_+=0}^{\epsilon=\pi}$ and $W_{k_+=\pi}^{\epsilon=0}$	$-2\nu_0^{(0)}\nu_+^{(0)}$	$S_- = \sin(\frac{\theta_x}{2})\sigma_x - \cos(\frac{\theta_x}{2})\sigma_z$

TABLE S2. **The detailed bulk-boundary correspondence (BBC) with varying topological invariants of outer region.** The topological variant of inner region is fixed as $\{-\frac{1}{2}, -\frac{1}{2}, -\frac{1}{2}\}$. Here the situations with square and diamond domain walls are represented by □ and ◇, respectively. In these two columns, chiral, corner, and unchiral indicate the appearance of chiral boundary states, corner states, and unchiral boundary states, respectively, while unchiral states may just locate at domain walls separating $x+y$ or $x-y$ direction, which has been marked out. The last four columns discuss the topological properties for certain 1D subspace. Here, 1 and −1 indicates the existence of degenerated boundary states with effective energy 0 and π, respectively. The results for $k_x = \pi$ ($k_x = -\frac{\pi}{2}$) and $k_y = 0, \pi$ ($k_y = \pm\frac{\pi}{2}$) subspaces is equivalent to $k_x = 0$ ($k_x = \frac{\pi}{2}$) subspace. And the degenerated boundary states with 0 (π) energy in $k_\pm \equiv k_x \pm k_y = 0$ subspace will correspond to the ones with π (0) energy in $k_\pm = \pi$ subspace.

$\{\nu_0^{(0)}, \nu_{\pi/2}^{(0)}, \nu_+^{(0)}\}$	□	◇	$k_x = 0$	$k_x = \frac{\pi}{2}$	$k_- = 0$	$k_+ = 0$
$\{\frac{1}{2}, -\frac{1}{2}, \frac{1}{2}\}$	chiral	chiral	±1	\	1	1
$\{\frac{1}{2}, -\frac{1}{2}, -\frac{1}{2}\}$	chiral	chiral	±1	\	−1	−1
$\{-\frac{1}{2}, \frac{1}{2}, \frac{1}{2}\}$	chiral	chiral	\	±1	1	−1
$\{-\frac{1}{2}, \frac{1}{2}, -\frac{1}{2}\}$	chiral	chiral	\	±1	−1	1
$\{\frac{1}{2}, \frac{1}{2}, \frac{1}{2}\}$	unchiral	unchiral ($x+y$)	±1	±1	±1	\
$\{\frac{1}{2}, \frac{1}{2}, -\frac{1}{2}\}$	unchiral	unchiral ($x-y$)	±1	±1	\	±1
$\{-\frac{1}{2}, -\frac{1}{2}, \frac{1}{2}\}$	corner	unchiral	\	\	±1	±1
$\{-\frac{1}{2}, -\frac{1}{2}, -\frac{1}{2}\}$	\	\	\	\	\	\

with

$$S_0 = \cos(\frac{\theta_x - \theta_y}{2})\sigma_x + \sin(\frac{\theta_x - \theta_y}{2})\sigma_z. \tag{S16}$$

The chiral symmetry operator can be transformed into σ_z: $R(\frac{\theta_x-\theta_y-\pi}{2})S_0R^{-1}(\frac{\theta_x-\theta_y-\pi}{2}) = \sigma_z$, indicating the effective Hamiltonian

will be off-diagonal under this transformation:

$$R(\frac{\theta_x-\theta_y-\pi}{2})H_{\rm F}(k_x,0)R^{-1}(\frac{\theta_x-\theta_y-\pi}{2}) = \frac{\arccos(h_0)}{\sqrt{1-h_0^2}}\begin{pmatrix} 0 & d_0(k_x) \\ d_0^*(k_x) & 0 \end{pmatrix}, \tag{S17}$$

where $d_0(k_x) = -\sin(k_x) - i\sin(\frac{\theta_x+\theta_y}{2})\cos(k_x)$. Then we compute the 1D winding number as:

$$\begin{aligned} \tilde{\nu}_0 &= \frac{1}{2\pi i}\int_{-\pi}^{\pi} dk_x \frac{d}{dk_x}\ln[\frac{\arccos(h_0)}{\sqrt{1-h_0^2}}d_0(k_x)] \\ &= -\mathrm{sign}[\sin(\frac{\theta_x+\theta_y}{2})]. \end{aligned} \tag{S18}$$

Here, it characterizes the number and polarization of boundary states with $k_y = 0$, satisfying $m_+ - m_- = \nu_{0,L} - \nu_{0,R}$, where $m_\pm$ is the number of boundary states with eigenvalues ± 1 of S_0 and $\nu_{0,L}$ ($\nu_{0,R}$) is the winding number for the left (right) side to domain wall [8].

However, this two-band Floquet system can exhibit both zero- and π-energy gaps, necessitating separate topological invariants $\nu_0^{(0)}$ and $\nu_0^{(\pi)}$ to characterize boundary states at each gap, i.e., $m_+^{(0/\pi)} - m_-^{(0/\pi)} = \nu_{0,L}^{(0/\pi)} - \nu_{0,R}^{(0/\pi)}$. To analyze this systematically, we define two auxiliary operators:

$$F_0 = R(\theta_y)T_x^{(-)}R(\frac{\theta_x-\theta_y}{2}), \quad G_0 = R^{-1}(\frac{\theta_x-\theta_y}{2})T_x^{(+)}R(\theta_x), \tag{S19}$$

with

$$T_x^{(-)} = \begin{pmatrix} 1 & 0 \\ 0 & e^{ik_x} \end{pmatrix}, \quad T_x^{(+)} = \begin{pmatrix} e^{-ik_x} & 0 \\ 0 & 1 \end{pmatrix}. \tag{S20}$$

Since $S_0F_0S_0G_0 = \sigma_0$, we have $S_0F_0S_0 = G_0^{-1}$ and $S_0G_0S_0 = F_0^{-1}$. Therefore, the product of G_0 and F_0 is chiral-symmetric:

$$\begin{aligned} S_0F_0G_0S_0 &= S_0F_0S_0S_0G_0S_0 = G_0^{-1}F_0^{-1}, \\ S_0G_0F_0S_0 &= S_0G_0S_0S_0F_0S_0 = F_0^{-1}G_0^{-1}. \end{aligned} \tag{S21}$$

Obviously, $F_0G_0 = U(k_x, 0)$ is the one we just discussed. We obtain the other one as:

$$\begin{aligned} U'(k_x,0) &= G_0F_0 = h_0\sigma_0 - i(h'_x\sigma_x + h'_y\sigma_y + h'_z\sigma_z), \\ h'_x &= -\cos(\frac{\theta_x+\theta_y}{2})\sin(\frac{\theta_x-\theta_y}{2})\sin(k_x), \\ h'_y &= \sin(\frac{\theta_x+\theta_y}{2}), \\ h'_z &= \cos(\frac{\theta_x+\theta_y}{2})\cos(\frac{\theta_x-\theta_y}{2})\sin(k_x), \end{aligned} \tag{S22}$$

Correspondingly, the effective Hamiltonian, $H'_{\rm F}(\mathbf{k}) = i\ln(U'(\mathbf{k}))$, is off-diagonal through the same transformation:

$$R(\frac{\theta_x-\theta_y-\pi}{2})H'_{\rm F}(k_x,0)R^{-1}(\frac{\theta_x-\theta_y-\pi}{2}) = \frac{\arccos(h_0)}{\sqrt{1-h_0^2}}\begin{pmatrix} 0 & d'_0(k_x) \\ d'^*_0(k_x) & 0 \end{pmatrix}, \tag{S23}$$

with $d'_0(k_x) = -\cos(\frac{\theta_x+\theta_y}{2})\sin(k_x) - i\sin(\frac{\theta_x+\theta_y}{2})$. The winding number is

$$\nu'_0 = \frac{1}{2\pi i}\int_{-\pi}^{\pi} dk_x \frac{d}{dk_x}\ln[\frac{\arccos(h_0)]}{\sqrt{1-h_0^2}}d'_0(k_x)] = 0 \tag{S24}$$

On the other hand, since $U(k_x, 0)$ and $U'(k_x, 0)$ are linked through an unitary transformation $U'(k_x, 0) = G_0U(k_x, 0)G_0^{-1}$, they have the same energy spectrum and the number of boundary states at each gap, $m_+^{(0/\pi)} + m_-^{(0/\pi)} = m_+'^{(0/\pi)} + m_-'^{(0/\pi)}$, but with distinct polarizations. To unveil it, we suppose there is a boundary states of $U(k_x, 0)$ with eigenvalue $\varepsilon \in \{0, \pi\}$: $U(k_x, 0)|\phi\rangle = e^{-i\varepsilon}|\phi\rangle$,

which will also be eigenstate of S_0: $S_0|\phi\rangle = e^{-i\gamma}|\phi\rangle$ with $\gamma \in \{0, \pi\}$. Therefore, $G_0|\phi\rangle$ will be eigenstate of $U'(k_x, 0)$ with same eigenvalue:

$$U'(k_x, 0)G_0|\phi\rangle = G_0 U(k_x, 0)G_0^{-1}G_0|\phi\rangle = e^{-i\varepsilon}G_0|\phi\rangle. \tag{S25}$$

But the state $G_0|\phi\rangle$ may be associated with eigenvalue of S_0 which is different from $|\phi\rangle$:

$$S_0 G_0|\phi\rangle = F_0^{-1}S_0|\phi\rangle = e^{-i\gamma}F_0^{-1}|\phi\rangle = e^{-i\gamma}F_0^{-1}G_0^{-1}G_0|\phi\rangle = e^{-i\gamma}U'^{-1}(k_x, 0)G_0|\phi\rangle = e^{-i(\gamma-\varepsilon)}G_0|\phi\rangle \tag{S26}$$

where the first equation is based on the fact: $S_0F_0S_0G_0 = \sigma_0$. The above two relations (S25) and (S26) indicate the polarization will be same for zero-energy boundary states of these two operator: $m_\pm^{(0)} = m_\pm'^{(0)}$, while the polarization will be opposite for π-energy boundary states: $m_\pm^{(\pi)} = m_\mp'^{(\pi)}$. To conclude, we can get following relations:

$$\begin{aligned} m_+^{(0)} - m_-^{(0)} &= \nu_{0,L}^{(0)} - \nu_{0,R}^{(0)} = \nu_{0,L}'^{(0)} - \nu_{0,R}'^{(0)} \implies \nu_0^{(0)} = \nu_0'^{(0)}, \\ m_+^{(\pi)} - m_-^{(\pi)} &= \nu_{0,L}^{(\pi)} - \nu_{\pi,R}^{(0)} = -[\nu_{0,L}'^{(\pi)} - \nu_{0,R}'^{(\pi)}] \implies \nu_0^{(\pi)} = -\nu_0'^{(\pi)}, \\ \nu_0^{(0)} + \nu_0^{(\pi)} &= \tilde{\nu}_0, \qquad \nu_0'^{(0)} + \nu_0'^{(\pi)} = \nu_0', \end{aligned} \tag{S27}$$

which leads to the winding number of $U(k_x, 0)$:

$$\{\nu_0^{(0)}, \nu_0^{(\pi)}\} = \frac{1}{2}\{\tilde{\nu}_0 + \nu_0', \tilde{\nu}_0 - \nu_0'\} = \frac{-\text{sign}[\sin(\frac{\theta_x+\theta_y}{2})]}{2}\{1, 1\}. \tag{S28}$$

This result indicates $\nu_0^{(0)}$ and $\nu_0^{(\pi)}$ change their values when $\sin(\frac{\theta_x+\theta_y}{2})$ changes its sign , which coincides with the simultaneous closing of the π- and 0-gaps of $U(k_x, 0)$ at $\theta_x + \theta_y = 2n\pi$. Due to $T_y(\pi) = -T_y(0)$, the winding number of $U(k_x, \pi)$ can be obtained by $\{\nu_\pi^{(0)}, \nu_\pi^{(\pi)}\} = \{\nu_0^{(\pi)}, \nu_0^{(0)}\}$. For the subspace with $k_x = 0, \pi$, $U(0/\pi, k_y)$ can be obtained by $R^{-1}(\theta_y)U(k_x, 0/\pi)R(\theta_y)$ and transformation $k_x \to k_y$, which suggests that $U(0/\pi, k_y)$ and $U(k_x, 0/\pi)$ will hold the same number of boundary states in each gap. More concretely, if $U(k_x, 0)|\phi\rangle = e^{-i\varepsilon}|\phi\rangle$, we will have $U(0/\pi, k_y)R^{-1}(\theta_y)|\phi\rangle = e^{-i\varepsilon}R^{-1}(\theta_y)|\phi\rangle$. Furthermore, $U(0/\pi, k_y)$ will hold a chiral symmetry

$$R^{-1}(\theta_y)S_0R(\theta_y) = \cos(\frac{\theta_x + \theta_y}{2})\sigma_x + \sin(\frac{\theta_x + \theta_y}{2})\sigma_z = S_{\pi/2}, \tag{S29}$$

which indicates that if $S_0|\phi\rangle = e^{-i\gamma}|\phi\rangle$, we will have $S_{\pi/2}R(\theta_y)|\phi\rangle = e^{-i\gamma}R(\theta_y)|\phi\rangle$. In summary, the associated winding number for $U(0/\pi, k_y)$ at (θ_x, θ_y) will also be $\{\nu_0^{(0)}, \nu_0^{(\pi)}\}$ given in Eq. (S28), but chiral symmetry operator becomes $S_{\pi/2} = \cos(\frac{\theta_x+\theta_y}{2})\sigma_x + \sin(\frac{\theta_x+\theta_y}{2})\sigma_z$, as given in Table S1, where we have defined the 1D winding number for ϵ-gap in the subspace $\boldsymbol{k}$ as $W_{\boldsymbol{k}}^{\epsilon}$.

Similarly, the winding number for $U(k_x, \pi/2)$ can also be obtained in this approach. The main procedure is listed as following. $U(k_x, \pi/2)$ also holds a chiral symmetry,

$$S_{\pi/2}U(k_x, \pi/2)S_{\pi/2} = U^{-1}(k_x, \pi/2) \tag{S30}$$

with the symmetry operator

$$S_{\pi/2} = \cos(\frac{\theta_x + \theta_y}{2})\sigma_x + \sin(\frac{\theta_x + \theta_y}{2})\sigma_z. \tag{S31}$$

Associated effective Hamiltonian can be transformed into off-diagonal form:

$$R(\frac{\theta_x + \theta_y - \pi/2}{2})H_{\mathrm{F}}(k_x, \pi/2)R^{-1}(\frac{\theta_x + \theta_y - \pi/2}{2}) = \frac{\arccos[h_0(k_x, \frac{\pi}{2})]}{\sqrt{1 - h_0^2(k_x, \frac{\pi}{2})}}\begin{pmatrix} 0 & h_{\pi/2}(k_x) \\ h_{\pi/2}^*(k_x) & 0 \end{pmatrix} \tag{S32}$$

with $h_{\pi/2}(k_x) = -\cos(k_x) + i\sin(\frac{\theta_x-\theta_y}{2})\sin(k_x)$. Thereby, associated winding number is given by:

$$\begin{aligned} \tilde{\nu}_{\pi/2} &= \frac{1}{2\pi i}\int_{-\pi}^{\pi} dk_x \frac{d}{dk_x}\ln[\frac{\arccos[h_0(k_x, \frac{\pi}{2})]}{\sqrt{1 - h_0^2(k_x, \frac{\pi}{2})}}d_{\pi/2}(k_x)] \\ &= -\text{sign}[\sin(\frac{\theta_x - \theta_y}{2})]. \end{aligned} \tag{S33}$$

And the associated auxiliary operators can be defined by:

$$F_{\pi/2} = -i\sigma_z R(\theta_y) T_x^{(-)} u(\pi/2, \vec{n}_\perp), \qquad G_{\pi/2} = u^{-1}(\pi/2, \vec{n}_\perp) T_x^{(+)} R(\theta_x), \tag{S34}$$

with $u(\pi/2, \vec{n}_\perp) = \exp\{-i\pi/4[\sin(\frac{\theta_x+\theta_y}{2})\sigma_x - \cos(\frac{\theta_x+\theta_y}{2})\sigma_z]\}$. Obviously, $U(k_x, \pi/2) = F_{\pi/2}G_{\pi/2}$, and $S_{\pi/2}F_{\pi/2}S_{\pi/2}G_{\pi/2} = \sigma_0$. Another chiral symmetric operator is given by

$$\begin{aligned}
U'(k_x, \pi/2) &= G_{\pi/2}F_{\pi/2} = h_0\sigma_0 - i(h'_x\sigma_x + h'_y\sigma_y + h'_z\sigma_z),\\
h'_x &= \sin(\frac{\theta_x+\theta_y}{2})[\sin(\frac{\theta_x+\theta_y}{2})\sin(\frac{\theta_x-\theta_y}{2}) - \cos(\frac{\theta_x+\theta_y}{2})\cos(\frac{\theta_x-\theta_y}{2})\cos(k_x)],\\
h'_y &= \cos(\frac{\theta_x+\theta_y}{2})\sin(\frac{\theta_x-\theta_y}{2}) + \sin(\frac{\theta_x+\theta_y}{2})\cos(\frac{\theta_x-\theta_y}{2})\cos(k_x),\\
h'_z &= -\cos(\frac{\theta_x+\theta_y}{2})[\sin(\frac{\theta_x+\theta_y}{2})\sin(\frac{\theta_x-\theta_y}{2}) - \cos(\frac{\theta_x+\theta_y}{2})\cos(\frac{\theta_x-\theta_y}{2})\cos(k_x)].
\end{aligned}\tag{S35}$$

After transformation, associated effective Hamiltonian will also be off-diagonal:

$$R(\frac{\theta_x+\theta_y-\pi/2}{2})H'_{\mathrm{F}}(k_x, \pi/2)R^{-1}(\frac{\theta_x+\theta_y-\pi/2}{2}) = \frac{\arccos(h_0)}{\sqrt{1-h_0^2}}\begin{pmatrix} 0 & d'_{\pi/2}(k_x)\\ d'^{*}_{\pi/2}(k_x) & 0\end{pmatrix} \tag{S36}$$

with $d'_{\pi/2}(k_x) = \sin(\frac{\theta_x+\theta_y}{2})\sin(\frac{\theta_x-\theta_y}{2}) - \cos(\frac{\theta_x+\theta_y}{2})\cos(\frac{\theta_x-\theta_y}{2})\cos(k_x) - i[\cos(\frac{\theta_x+\theta_y}{2})\sin(\frac{\theta_x-\theta_y}{2}) + \sin(\frac{\theta_x+\theta_y}{2})\cos(\frac{\theta_x-\theta_y}{2})\cos(k_x)]$. Corresponding 1D winding number will be

$$\nu'_{\pi/2} = \frac{1}{2\pi i}\int_{-\pi}^{\pi} dk_x \frac{d}{dk_x}\ln[\frac{\arccos(h_0)}{\sqrt{1-h_0^2}}d'_{\pi/2}(k_x)] = 0. \tag{S37}$$

Thereby, the winding number $\nu_{\pi/2}^{(0)}$ and $\nu_{\pi/2}^{(\pi)}$, which characterize the edge states of $U(k_x, \pi/2)$ at 0 and π gap respectively, are given by

$$\{\nu_{\pi/2}^{(0)}, \nu_{\pi/2}^{(\pi)}\} = \frac{1}{2}\{\tilde{\nu}_{\pi/2} + \nu'_{\pi/2}, \tilde{\nu}_{\pi/2} - \nu'_{\pi/2}\} = \frac{-\mathrm{sign}[\sin(\frac{\theta_x-\theta_y}{2})]}{2}\{1, 1\}, \tag{S38}$$

according to the fact: the π- and 0-gaps of $U(k_x, \pi/2)$ close simultaneously when $\theta_x - \theta_y = 2n\pi$. And the winding number for $U(k_x, -\pi/2)$ will be given by $\{\nu_{-\pi/2}^{(0)}, \nu_{-\pi/2}^{(\pi)}\} = \{\nu_{\pi/2}^{(\pi)}, \nu_{\pi/2}^{(0)}\}$, considering $T_y(-\pi/2) = -T_y(\pi/2)$. In addition, $U(\pm\pi/2, k_y)$ can be obtained by $U(\pm\pi/2, k_y) = R(\theta_y)U(k_x, \pm\pi/2)R^{-1}(\theta_y)$ and transformation $k_x \to k_y$. Suggesting $U(\pm\pi/2, k_y)$ and $U(k_x, \pm\pi/2)$ will hold same number of boundary states at each gap: if $U(k_x, \pm\pi/2)|\phi\rangle = e^{-i\varepsilon}|\phi\rangle$, $U(\pm\pi/2, k_y)R(\theta_y)|\phi\rangle = e^{-i\varepsilon}R(\theta_y)|\phi\rangle$. And $U(\pm\pi/2, k_y)$ will hold a chiral symmetry

$$R(\theta_y)S_{\pi/2}R^{-1}(\theta_y) = \cos(\frac{\theta_x-\theta_y}{2})\sigma_x + \sin(\frac{\theta_x-\theta_y}{2})\sigma_z = S_0 \tag{S39}$$

And associated polarization will be same, i.e., if $S_{\pi/2}|\phi\rangle = e^{-i\gamma}|\phi\rangle$, then $S_0R(\theta_y)|\phi\rangle = e^{-i\gamma}R(\theta_y)|\phi\rangle$. So associated winding number for $U(\pm\pi/2, k_y)$ will also be $\{\nu_{\pi/2}^{(0)}, \nu_{\pi/2}^{(\pi)}\}$ given in Eq. (S38) but chiral symmetry operator being $S_0 = \cos(\frac{\theta_x-\theta_y}{2})\sigma_x + \sin(\frac{\theta_x-\theta_y}{2})\sigma_z$, as given in Table S1.

For the operator U, there are several topological invariants yet: Rudner-Lindner-Berg-Levin winding numbers $W[U]$ used in Refs. [3, 4], and 1D winding numbers defined above: $\nu_0^{(0/\pi)}$ and $\nu_{\pi/2}^{(0/\pi)}$. While they are not independent: that is $\nu_0^{(0)} = \nu_0^{(\pi)}$ and $\nu_{\pi/2}^{(0)} = \nu_{\pi/2}^{(\pi)}$; as well as $W[U] = \tilde{\nu}_0\tilde{\nu}_{\pi/2} = 4\nu_0^{(0)}\nu_{\pi/2}^{(0)}$. Thereby, we can just use $\nu_0^{(0)}$ and $\nu_{\pi/2}^{(0)}$, which can characterize topological phases with 1D boundary states under square domain walls. We note that while $W[U]$ takes period 2π of parameters θ_x and θ_y, $\nu_0^{(0)}$ take period 4π of them. And these two topological invariants will determine the form of boundary states, as given in Table I of main text and discussed following.

Two sets of chiral boundary states will appear at each gap when either $\nu_0^{(0)}$ or $\nu_{\pi/2}^{(0)}$ is different for inner and outer regions, and their chirality will be determined by the difference of $\nu_0^{(0)}\nu_{\pi/2}^{(0)}$ of two regions. Furthermore, the energy spectra of chiral boundary states will cross at $k_y = 0, \pi$ when $\nu_0^{(0)}$ is different for two regions, and it will cross at $k_y = \pm\pi/2$ when $\nu_{\pi/2}^{(0)}$ is different. Otherwise, when both of $\nu_0^{(0)}, \nu_{\pi/2}^{(0)}$ are different for two regions, wrapped (unchiral) boundary states can appear with crossing

points at $k_y = 0, \pm\pi/2, \pi$. Finally, when two regions take the same values of $\nu_0^{(0)}$ and $\nu_{\pi/2}^{(0)}$, 1D boundary state will vanish under square domain walls. Notably, as displayed in Fig. 2c of main text, 0D corner states may appear for this situation, which is generated by the 1D boundary states under diamond domain walls. Thereby, it is necessary to investigate the topological properties of 1D subspaces $k_\pm = 0, \pi$.

To proceed, we rewrite Eq. (S2) into:

$$\begin{aligned} U(\mathbf{k}) &= h_0\sigma_0 - i(h_x\sigma_x + h_y\sigma_y + h_z\sigma_z), \\ h_0 &= \cos(\frac{\theta_x}{2})\cos(\frac{\theta_y}{2})\cos(k_+) - \sin(\frac{\theta_x}{2})\sin(\frac{\theta_y}{2})\cos(k_-), \\ h_x &= -\sin(\frac{\theta_x}{2})\cos(\frac{\theta_y}{2})\sin(k_+) + \cos(\frac{\theta_x}{2})\sin(\frac{\theta_y}{2})\sin(k_-), \\ h_y &= \sin(\frac{\theta_x}{2})\cos(\frac{\theta_y}{2})\cos(k_+) + \cos(\frac{\theta_x}{2})\sin(\frac{\theta_y}{2})\cos(k_-), \\ h_z &= \cos(\frac{\theta_x}{2})\cos(\frac{\theta_y}{2})\sin(k_+) + \sin(\frac{\theta_x}{2})\sin(\frac{\theta_y}{2})\sin(k_-), \end{aligned} \tag{S40}$$

Similar to the discussions in Supplementary Note 1, a chiral symmetry

$$S_+U(k_+, k_- = 0)S_+ = U^{-1}(k_+, k_- = 0) \tag{S41}$$

appears for the 1D subspace $k_- = 0$ with $S_+ = \cos(\frac{\theta_x}{2})\sigma_x + \sin(\frac{\theta_x}{2})\sigma_z$. It can be transformed to σ_z by:

$$R(\frac{\theta_x - \pi}{2})S_+R^{-1}(\frac{\theta_x - \pi}{2}) = \sigma_z \tag{S42}$$

and the effective Hamiltonian becomes

$$R(\frac{\theta_x - \pi}{2})H_{\mathrm{F}}R^{-1}(\frac{\theta_x - \pi}{2}) = \frac{\arccos(h_0)}{\sqrt{1 - h_0^2}}\begin{pmatrix} 0 & d_+ \\ d_+^* & 0 \end{pmatrix} \tag{S43}$$

with $d_+ = -\cos(\frac{\theta_y}{2})\sin(k_+) - i[\sin(\frac{\theta_x}{2})\cos(\frac{\theta_y}{2})\cos(k_+) + \cos(\frac{\theta_x}{2})\sin(\frac{\theta_y}{2})]$. And the corresponding winding number will be

$$\begin{aligned} \tilde{\nu}_+ &= -\mathrm{sign}[\sin(\frac{\theta_x}{2})]\frac{1 + \mathrm{sign}[|\sin(\frac{\theta_x}{2})\cos(\frac{\theta_y}{2})| - |\cos(\frac{\theta_x}{2})\sin(\frac{\theta_y}{2})|]}{2} \\ &= \mathrm{sign}[\sin(\frac{\theta_x}{2})]\frac{\mathrm{sign}[\cos(\theta_x) - \cos(\theta_y)] - 1}{2}. \end{aligned} \tag{S44}$$

To obtain winding number $\nu_+^{(0)}$ and $\nu_+^{(\pi)}$, we note that in this 1D subspace, $U(k_+, k_- = 0) = e^{-i\frac{k_+}{2}\sigma_z}R(\theta_y)e^{-i\frac{k_+}{2}\sigma_z}R(\theta_x)$. And it can be changed into another operator:

$$U_{1,+} = R(\frac{\theta_x}{2})U(k_+, k_- = 0)R^{-1}(\frac{\theta_x}{2}) = R(\frac{\theta_x}{2})e^{-i\frac{k_+}{2}\sigma_z}R(\theta_y)e^{-i\frac{k_+}{2}\sigma_z}R(\frac{\theta_x}{2}). \tag{S45}$$

Obviously, $U_{1,+}$ holds the same winding number as $U(k_+, k_- = 0)$ and supports a chiral symmetry read as

$$R(\frac{\theta_x}{2})S_+R^{-1}(\frac{\theta_x}{2}) = \sigma_x. \tag{S46}$$

Based on these, we can define two auxiliary operators:

$$F_+ = R(\frac{\theta_x}{2})e^{-i\frac{k_+}{2}\sigma_z}R(\frac{\theta_y}{2}), \qquad G_+ = R(\frac{\theta_y}{2})e^{-i\frac{k_+}{2}\sigma_z}R(\frac{\theta_x}{2}), \tag{S47}$$

which $\sigma_xF_+\sigma_x = G_+^{-1}$ and $U_{1,+} = F_+G_+$. In addition,

$$U'_{1,+} = G_+F_+ = R(\frac{\theta_y}{2})e^{-i\frac{k_+}{2}\sigma_z}R(\theta_x)e^{-i\frac{k_+}{2}\sigma_z}R(\frac{\theta_y}{2}) \tag{S48}$$

also holds chiral symmetry with operator σ_x. After comparing $U_{1,+}$ and $U'_{1,+}$ ($\theta_x \leftrightarrow \theta_y$), we can get the winding number of $U'_{1,+}$

directly:

$$\nu'_{+} = \text{sign}[\sin(\frac{\theta_y}{2})]\frac{\text{sign}[\cos(\theta_y) - \cos(\theta_x)] - 1}{2}. \tag{S49}$$

Combining the above information, we can get the winding numbers of $U(k_+, k_- = 0)$

$$\{\nu_+^{(0)}, \nu_+^{(\pi)}\} = \frac{1}{2}\{\tilde{\nu}_+ + \nu'_+, \tilde{\nu}_+ - \nu'_+\}. \tag{S50}$$

For the subspace $k_- = \pi$, we find

$$U(k_+, k_- = \pi) = e^{-i\frac{k_+}{2}\sigma_z}e^{i\frac{\pi}{2}\sigma_z}R(\theta_y)e^{-i\frac{\pi}{2}\sigma_z}e^{-i\frac{k_+}{2}\sigma_z}R(\theta_x) = e^{-i\frac{k_+}{2}\sigma_z}R(-\theta_y)e^{-i\frac{k_+}{2}\sigma_z}R(\theta_x) \tag{S51}$$

And then these winding numbers defined through Eq. (S49) are also useful for this subspace after $\theta_y \to -\theta_y$. That is the winding numbers defined in Eq. (S50) should be changed their status for $k_- = \pi$ subspace: $\nu_+^{(0)} \leftrightarrow \nu_+^{(\pi)}$.

Similarly, for the subspace $k_+ = 0$, another chiral symmetry

$$S_- U(k_+ = 0, k_-)S_- = U^{-1}(k_+ = 0, k_-) \tag{S52}$$

appears with $S_- = \sin(\frac{\theta_x}{2})\sigma_x - \cos(\frac{\theta_x}{2})\sigma_z$. It can be transformed to σ_z by:

$$R(\frac{\theta_x + 2\pi}{2})S_- R^{-1}(\frac{\theta_x + 2\pi}{2}) = \sigma_z \tag{S53}$$

and the effective Hamiltonian becomes

$$R(\frac{\theta_x + 2\pi}{2})H_{\text{F}}R^{-1}(\frac{\theta_x + 2\pi}{2}) = \frac{\arccos(h_0)}{\sqrt{1 - h_0^2}}\begin{pmatrix} 0 & d_- \\ d_-^* & 0 \end{pmatrix} \tag{S54}$$

with $d_- = -\sin(\frac{\theta_y}{2})\sin(k_-) - i[\cos(\frac{\theta_x}{2})\sin(\frac{\theta_y}{2})\cos(k_-) + \sin(\frac{\theta_x}{2})\cos(\frac{\theta_y}{2})]$. And the corresponding winding number is

$$\begin{aligned} \tilde{\nu}_- &= -\text{sign}[\cos(\frac{\theta_x}{2})]\frac{1 + \text{sign}[|\cos(\frac{\theta_x}{2})\sin(\frac{\theta_y}{2})| - |\sin(\frac{\theta_x}{2})\cos(\frac{\theta_y}{2})|]}{2} \\ &= \text{sign}[\cos(\frac{\theta_x}{2})]\frac{\text{sign}[\cos(\theta_y) - \cos(\theta_x)] - 1}{2}. \end{aligned} \tag{S55}$$

And in this 1D subspace, $U(k_+ = 0, k_-) = e^{i\frac{k_-}{2}\sigma_z}R(\theta_y)e^{-i\frac{k_-}{2}\sigma_z}R(\theta_x)$, changing into another operator:

$$U_{1,-} = R(\frac{\theta_x}{2})U(k_+, k_- = 0)R^{-1}(\frac{\theta_x}{2}) = R(\frac{\theta_x}{2})e^{i\frac{k_-}{2}\sigma_z}R(\theta_y)e^{-i\frac{k_-}{2}\sigma_z}R(\frac{\theta_x}{2}) \tag{S56}$$

whose chiral symmetry operator is $-\sigma_z$.

Based on these, we can define two auxiliary operators:

$$F_- = R(\frac{\theta_x}{2})e^{i\frac{k_-}{2}\sigma_z}R(\frac{\theta_y}{2}), \qquad G_- = R(\frac{\theta_y}{2})e^{-i\frac{k_-}{2}\sigma_z}R(\frac{\theta_x}{2}), \tag{S57}$$

which $(-\sigma_z)F_-(-\sigma_z) = G_-^{-1}$ and $U_{1,-} = F_- G_-$. In addition,

$$U'_{1,-} = G_- F_- = R(\frac{\theta_y}{2})e^{-i\frac{k_-}{2}\sigma_z}R(\theta_x)e^{i\frac{k_-}{2}\sigma_z}R(\frac{\theta_y}{2}) \tag{S58}$$

also holds chiral symmetry with operator $-\sigma_z$. After comparing $U_{1,-}$ and $U'_{1,-}$ ($\theta_x \leftrightarrow \theta_y$, $k_- \to -k_-$), we can get the winding number of $U'_{1,+}$ directly:

$$\nu'_{-} = \text{sign}[\cos(\frac{\theta_y}{2})]\frac{1 - \text{sign}[\cos(\theta_x) - \cos(\theta_y)]}{2}. \tag{S59}$$

Combining this information, we can get the winding number of $U(k_+ = 0, k_-)$

$$\{\nu_-^{(0)}, \nu_-^{(\pi)}\} = \frac{1}{2}\{\tilde{\nu}_- + \nu'_-, \tilde{\nu}_- - \nu'_-\}. \tag{S60}$$

For the subspace $k_+ = \pi$, we have

$$U(k_+ = \pi, k_-) = e^{i\frac{k_-}{2}\sigma_z} e^{i\frac{\pi}{2}\sigma_z} R(\theta_y) e^{i\frac{\pi}{2}\sigma_z} e^{-i\frac{k_-}{2}\sigma_z} R(\theta_x) = -e^{i\frac{k_-}{2}\sigma_z} R(-\theta_y) e^{-i\frac{k_-}{2}\sigma_z} R(\theta_x) \tag{S61}$$

Thereby, these winding numbers are useful for this subspace after $\theta_y \to -\theta_y$ and gap $0 \leftrightarrow \pi$. After exchanging the winding number defined in Eq. (S60): $[\nu_-^{(0)} \leftrightarrow \nu_-^{(\pi)}]$, the winding number for $k_+ = \pi$ subspace is also obtained.

According to definitions of $\{\nu_-^{(0)}, \nu_-^{(\pi)}\}$ $[\{\nu_+^{(0)}, \nu_+^{(\pi)}\}]$, these invariants characterize the boundary states along diagonal (off-diagonal) direction with momentum $k_- = 0/\pi$ ($k_+ = 0/\pi$). However, there are too many topological invariants, so we need to find their underlying relations. Here, we find the relation between $\nu_\pm^{(0)}$ and $\nu_\pm^{(\pi)}$

$$\nu_+^{(\pi)} = 4\nu_0^{(0)}\nu_{\pi/2}^{(0)}\nu_+^{(0)}, \quad \nu_-^{(\pi)} = -4\nu_0^{(0)}\nu_{\pi/2}^{(0)}\nu_-^{(0)}, \tag{S62}$$

and the relation between $\nu_+^{(0)}$ and $\nu_-^{(0)}$:

$$\nu_-^{(0)} = 2\nu_{\pi/2}^{(0)}\nu_+^{(0)}, \tag{S63}$$

thereby, we can just use $\nu_+^{(0)}$ accompanied with $\nu_0^{(0)}$ and $\nu_{\pi/2}^{(0)}$ to compose a triplet topological invariant. Other topological invariants, including the Rudner-Lindner-Berg-Levin winding number and 1D winding number about other gap and/or other subspaces, can be obtained through $\nu_0^{(0)}$, $\nu_{\pi/2}^{(0)}$ and $\nu_+^{(0)}$, as shown in Table S1. With this triplet topological invariant, the parameter plane $\{\theta_x, \theta_y\}$ will be divided into eight distinct topological phases, as shown in Fig. 1 of the main text. And a more detailed BBC principle is provided in Table S2. **The notation of $\{\nu_0^{(0)}, \nu_{\pi/2}^{(0)}, \nu_+^{(0)}\}$ is simplified to $\{\nu_0, \nu_{\pi/2}, \nu_+\}$ in the main text.**

Supplementary Note 3. DISCUSSION ABOUT THE DISPERSION OF BOUNDARY STATES

In this section, we will provide the dispersion E-k, which can confirm the BBC and deepen our understanding of dynamical process. In Supplementary Note 3 A, we will calculate the dispersion E-k_x and E-k_y, associated with the dynamical process with square domain walls. Then we will discuss the dispersion E-k_+ and E-k_- in Supplementary Note 3 B, associated with the dynamical process with diamond domain walls.

A. Dispersion under square domain walls

In Fig. S1, we provide the spectra E-k_x and E-k_y with parameters used in Fig. 2 of main text. Here, we can confirm the BBC given in Table I of main text with more detail. Fig. S1a depicts the phase diagram with more detailed parameters. Fig. S1b gives the spectra for strong topological phases, where the chiral boundary states can be observed. For the parameters used here, $\nu_0^{(0)}$ is same while $\nu_{\pi/2}^{(0)}$ is different for two regions. Thus, the chiral boundary states will be degenerated with 0- and π−energies in $k_{x/y} = \pm\pi/2$ subspaces, which agrees with the prediction in Table I of main text.

In Fig. S1c, all of $\nu_0^{(0)}$, $\nu_{\pi/2}^{(0)}$ and $\nu_+^{(0)}$ are different for two regions. And in Fig. S1d, $\nu_0^{(0)}$ and $\nu_{\pi/2}^{(0)}$ are different while $\nu_+^{(0)}$ is same for two regions. Both of these two cases correspond to the weak topological phases with unchiral boundary states, which will be degenerated with 0 and π energies both in $k_{x/y} = 0, \pm\pi/2, \pi$ subspaces, i.e., these two phases hold similar dispersions under square domain walls and result in similar dynamical processes as shown in Fig. 4b of main text and Fig .S9e. The influences of invariants $\nu_+^{(0)}$ for weak topological phases will be reflected by the cases with diamond domain walls.

Finally, there are no 1D boundary states under square domain walls for the 2nd-order topological phases where $\nu_0^{(0)}$ and $\nu_{\pi/2}^{(0)}$ are same while $\nu_+^{(0)}$ is different for two regions, as shown in Fig. S1e. However, it does not indicate these phases is trivial. As shown in Fig. 2c, f of main text, 0D (1D) boundary states will appear for square (diamond) domain walls.

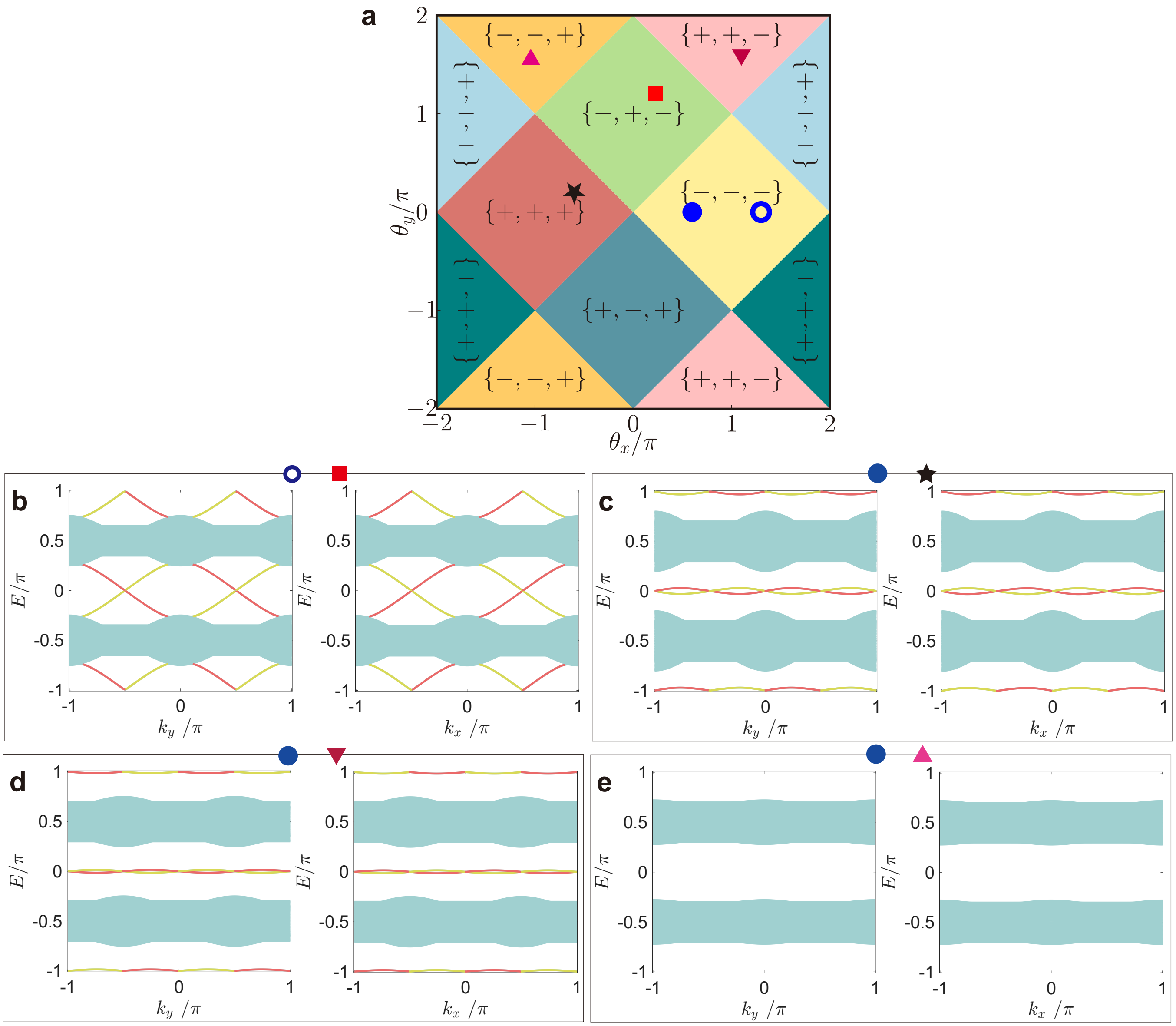

FIG. S1. **Spectra of effective Hamiltonian of U with domain walls paralleling along y- or x-direction. a** Phase diagram, where the symbols ○, ●, ■, ★, ▼, and ▲ denote the parameter sets $(\theta_x, \theta_y) = (1.3\pi, 0)$, $(0.6\pi, 0)$, $(0.3\pi, 1.2\pi)$, $(1.1\pi, 1.6\pi)$, and $(-1.042\pi, 1.6\pi)$, respectively. **b-e** Spectra, where ○ and ● denote the parameters inside the domain wall $\boldsymbol{\theta}^{\text{in}}$, and ■, ★, ▼, and ▲ denote the parameters outside the domain wall $\boldsymbol{\theta}^{\text{out}}$. The red (yellow) lines are the boundary states at right (left) or up (below) domain wall.

B. Dispersion under diamond domain walls

In this subsection, we discuss the dispersion E-$k_\pm$, which determines the dynamical process under diamond domain walls. However, $U(k_+, k_-) = e^{ik_-/2\sigma_z}e^{-ik_+/2\sigma_z}R(\theta_y)e^{-ik_+/2\sigma_z}e^{-ik_-/2\sigma_z}R(\theta_x)$, which involves $k_\pm/2$, seems questionable when we transform k_+ or k_- into real space, because these terms indicate the hopping across half-site. For the condition focusing on the real space along $x + y$, we evaluate the effect of operator $S_+ = e^{ik_+/2\sigma_z}R(\theta_y)e^{-ik_+/2\sigma_z}$. Thereby,

$$S_+|\uparrow_n\rangle = \cos\frac{\theta_{y,n+1/2}}{2}|\uparrow_n\rangle + \sin\frac{\theta_{y,n+1/2}}{2}|\downarrow_{n+1}\rangle \tag{S64}$$

$$S_+|\downarrow_n\rangle = -\sin\frac{\theta_{y,n-1/2}}{2}|\uparrow_{n-1}\rangle + \cos\frac{\theta_{y,n-1/2}}{2}|\downarrow_n\rangle \tag{S65}$$

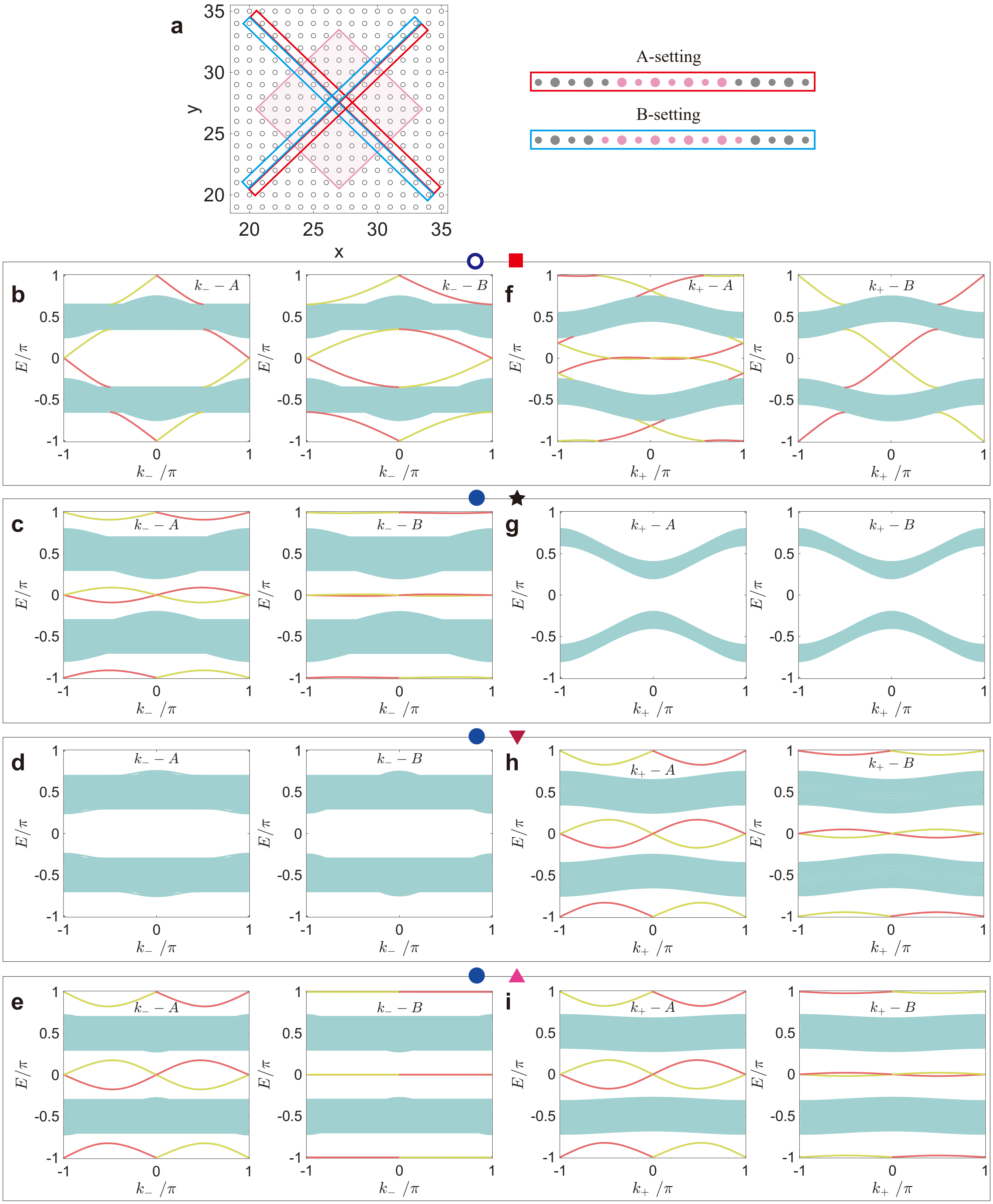

FIG. S2. **Spectra of effective Hamiltonian of U with domain walls parallel to $x+y$- or $x-y$-direction. a** The corresponding relationship between strips under diamond domain wall and their 1D settings. Here, the larger (smaller) circles denote main (auxiliary) sites, and pink (gray) color marks the inner (outer) region. **b-e** The dispersion of U with a domain wall separating $x+y$ direction, defined in Eq. (S66). **f-i** The dispersion of U with a domain wall separating $x-y$ direction, defined in Eq. (S67). Here, the yellow (red) lines are the boundary states around the domain wall with larger (smaller) value of $x \pm y$. The icons of parameter are same, as Fig. S1.

with n being the index of site along $x+y$-direction. As a result, QW operator U with good quantum number k_- and real space along $x+y$ direction will be

$$\begin{aligned} U(k_-) &= e^{ik_-/2\sigma_z}T_+S_+e^{-ik_-/2\sigma_z}R(\theta_x), \\ S_+ &= \sum_{n=1}^{N}(\cos\frac{\theta_{y,n+1/2}}{2}|\uparrow_n\rangle+\sin\frac{\theta_{y,n+1/2}}{2}|\downarrow_{n+1}\rangle)\langle\uparrow_n| + (-\sin\frac{\theta_{y,n-1/2}}{2}|\uparrow_{n-1}\rangle+\cos\frac{\theta_{y,n-1/2}}{2}|\downarrow_n\rangle)\langle\downarrow_n|, \\ T_+ &= \sum_n |\uparrow_{n+1}\rangle\langle\uparrow_n| + |\downarrow_{n-1}\rangle\langle\downarrow_n|. \end{aligned} \tag{S66}$$

Similarly, U with good quantum number k_- and real space along $x+y$ direction will be

$$\begin{aligned} U(k_+) &= e^{ik_+/2\sigma_z}S_-e^{-ik_+/2\sigma_z}R(\theta_x), \\ S_- &= \sum_{m=1}^{N}(\cos\frac{\theta_{y,m+1/2}}{2}|\uparrow_m\rangle+\sin\frac{\theta_{y,m+1/2}}{2}|\downarrow_{m+1}\rangle)\langle\uparrow_m| + (-\sin\frac{\theta_{y,m-1/2}}{2}|\uparrow_{m-1}\rangle+\cos\frac{\theta_{y,m-1/2}}{2}|\downarrow_m\rangle)\langle\downarrow_m|, \end{aligned} \tag{S67}$$

with m being the index of site along $x-y$-direction. Obviously, both of these two operators only involve the hopping between integer sites called main sites, but the value of θ_y in $S_\pm$ involves "half" sites called auxiliary sites. Considering the inversion symmetry, there are two distinct 1D chain settings. In the chain A (B) setting, the auxiliary sites between inner and outer regions belong to the outer (inner) region, as shown in Fig. S2a.

Actually, these two settings describes different stripes under the diamond geometry. Considering the coin operator $R(\theta_y)$ is acted after shift operator T_x [$U = T_yR(\theta_y)T_xR(\theta_x)$], the $n+1/2$ ($n-1/2$) auxiliary site actually is the right (left) site to n main site. Thereby during the state hopping across the main sites between two sites of domain wall, $R(\theta_y)$ is acted in the auxiliary site belong to the outer (inner) region for the stripes with even (odd) value of $x+y$ indexed by A (B) in Fig. S2a, establishing corresponding relationship to chain A (B) setting.

As displayed in Fig. S2, the appearance of boundary states both of these two settings can be expected by triplet topological invariants. Specifically, the topological invariant $\nu^{(0)}_{\pi/2}$ is different while $\nu^{(0)}_0$ and $\nu^{(0)}_+$ are same for two regions in Figs. S2b, f. Thereby there are no zero-energy boundary states for $k_+ = 0$ subspace, and according to the relation between $k_+ = 0$ and $k_+ = \pi$ subspace, there are also no π-energy boundary states in $k_+ = \pi$ subspace. However, according to the relation given in Eqs. (S62) and (S63), $\nu^{(\pi)}_+$ and $\nu^{(0)}_-$ will be different and $\nu^{(\pi)}_-$ will be same for two regions. As a result, there are 0-energy boundary states in $k_+ = \pi$ and $k_- = 0$ subspaces, as well as π-energy boundary states in $k_+ = 0$ and $k_- = \pi$ subspaces.

In addition, these boundary states show clockwise chirality associated with the value of $\nu^{(0)}_0\nu^{(0)}_{\pi/2}$. However, the group velocity is different for distinct setting. Especially, the velocity of boundary states $k_+ = 0$ or $k_+ = \pi$ is near zero (considerable) for A (B) setting as shown in Fig. S2f. For other cases involving two regions with different value of $\nu^{(0)}_0$ and $\nu^{(0)}_{\pi/2}$, unchiral boundary states will appear. In Fig. S2c, where $\nu^{(0)}_+$ are also different between two regions, unchiral boundary states will be degenerated with 0- and π-energy both in $k_+ = 0$ and $k_+ = \pi$ subspaces. But according to the relation in Eqs. (S62) and (S63), there is no boundary states in $k_- = 0$ and $k_- = \pi$ subspaces as shown in Figs. S2g. Similarly, 0- and π-energy unchiral boundary states appear both in $k_- = 0$ and $k_- = \pi$ subspaces instead of $k_+ = 0$ and $k_+ = \pi$ subspaces, when $\nu^{(0)}_+$ of two regions are same for the weak topological phases as shown in Figs. S2(d1-d2). Finally, 0- and π-energy unchiral boundary states appear in all of these for subspaces when $\nu^{(0)}_0$ and $\nu^{(0)}_{\pi/2}$ are same while $\nu^{(0)}_+$ is different for between two regions as shown in Figs. S2d, h. However, A and B setting can hold more different spectra for unchiral boundary states cases. Especially, the boundary states show opposite velocities under A and B setting in the cases given in Figs. S2h and S2i. Considering these two settings correspond to distinct strips under diamond geometry, the difference of spectra can be shown by putting initial states at different location in the dynamical process. It may be necessary to add information that during the system's evolution, the parity of $x+y$ remains invariant after integer periods, and thus no transition occurs between two settings. In addition, it should also be clarified that, in the evolution described in the main text, which setting and parity of $x+y$ of the initial state corresponds to.

Supplementary Note 4. DETAILS OF EXPERIMENTS

In this section, we introduce the details of our experiments. In Supplementary Note 4 A, we introduce the mapping between the time-bin sequence of light pulses and our simulated 2D lattice. In Supplementary Note 4 B, we present the detailed experimental realization of Floquet lattice in the main text. In Supplementary Note 4 C, we explain the methodology for creating and controlling domain walls in practical experimental settings. In Supplementary Note 4 D, we present our preparation of the pulse's initial states for dynamics, with implementations in both theory and experiment.

A. Mapping 2D lattice to 1D synthetic lattice in the time domain

Fig. S3 illustrates the mapping from the 2D lattice (x, y) to a 1D lattice t (in time domain), achieved by sequentially concatenating each row of sites in the ascending order of y. Specifically, our experimental system maps the 2D lattice onto a 1D temporal sequence by encoding each 53-site row as 53 consecutive time bins (13.99 ns spacing), with 755.50 ns intervals between corresponding sites in adjacent rows. We employ a 4 km single-mode fiber (SMF) delay line, which provides sufficient propagation delay to prevent temporal overlap between pulses at different time steps.

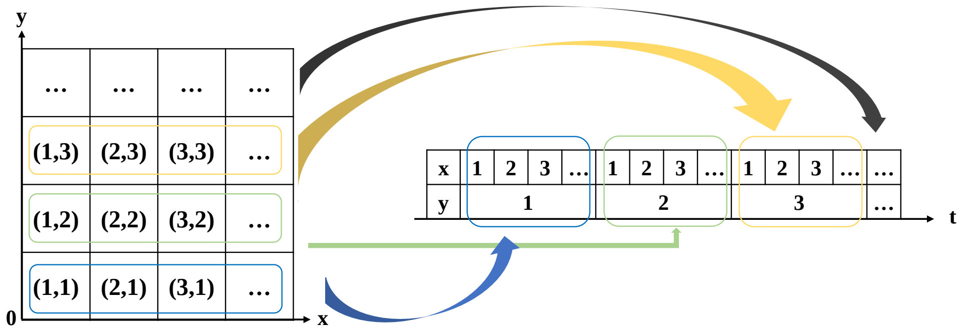

FIG. S3. Mapping the 2D lattice $(x,\ y)$ to 1D synthetic lattice in the time domain.

B. Detailed experimental realization of Floquet lattice

Fig. S4 presents the details of the experimental implementation of the unitary operations $U_x = T_x R(\theta_x)$ and $U_y = T_y R(\theta_y)$.

As an illustrative example, we describe the experimental implementation of the unitary operation R_1, where the laser pulse propagates sequentially through a quarter wave plate (QWP), an electro-optic modulator (EOM), another QWP and a half wave plate (HWP). The cumulative operation of this optical sequence is described by

$$R(\theta_x) = U_{\mathrm{HWP}}(\phi_1) U_{\mathrm{QWP@45^\circ}} U_{\mathrm{EOM}} U_{\mathrm{QWP@45^\circ}}. \tag{S68}$$

With

$$U_{\mathrm{HWP}}(\phi_1) = e^{-i\pi/2} \begin{bmatrix} \cos(2\phi_1) & \sin(2\phi_1) \\ \sin(2\phi_1) & -\cos(2\phi_1) \end{bmatrix}, \tag{S69}$$

$$U_{\mathrm{QWP@45^\circ}} = \frac{1}{\sqrt{2}} \begin{bmatrix} 1 & -i \\ -i & 1 \end{bmatrix}, \tag{S70}$$

$$U_{\mathrm{EOM}}(\phi_2) = \begin{bmatrix} e^{i2\phi_2} & 0 \\ 0 & -e^{-i2\phi_2} \end{bmatrix}, \tag{S71}$$

we have

$$R(\theta_x) = e^{-i\pi} \begin{bmatrix} \cos 2(\phi_1 - \phi_2) & -\sin 2(\phi_1 - \phi_2) \\ \sin 2(\phi_1 - \phi_2) & \cos 2(\phi_1 - \phi_2) \end{bmatrix}, \tag{S72}$$

where $\theta_x = 4(\phi_1 - \phi_2)$.

We provide details of the small optical loop containing the EOM. The light pulse enters the loop through a fiber polarizing beam splitter (FPBS), which splits it into two orthogonal polarization components. By positioning the EOM away from the loop's center, we ensure that the two components arrive at the EOM at distinct times, allowing independent phase control via synchronized nanosecond-scale electric pulses. After modulation, the components recombine at the FPBS and exit the loop with a well-defined relative phase $4\phi_2$.

The hopping operator T_x and T_y are implemented using an interferometric design comprising two polarization beam splitters (PBSs) and single-mode fibers (SMFs). Upon entering the first PBS, the laser pulse is split into two orthogonally polarized components. One component propagates through an SMF, while the other travels through free space, creating a relative delay between the two paths. The fiber lengths are precisely set to 5.60 m for T_x and and 151.10 m for T_y, as shown in Fig. S4.

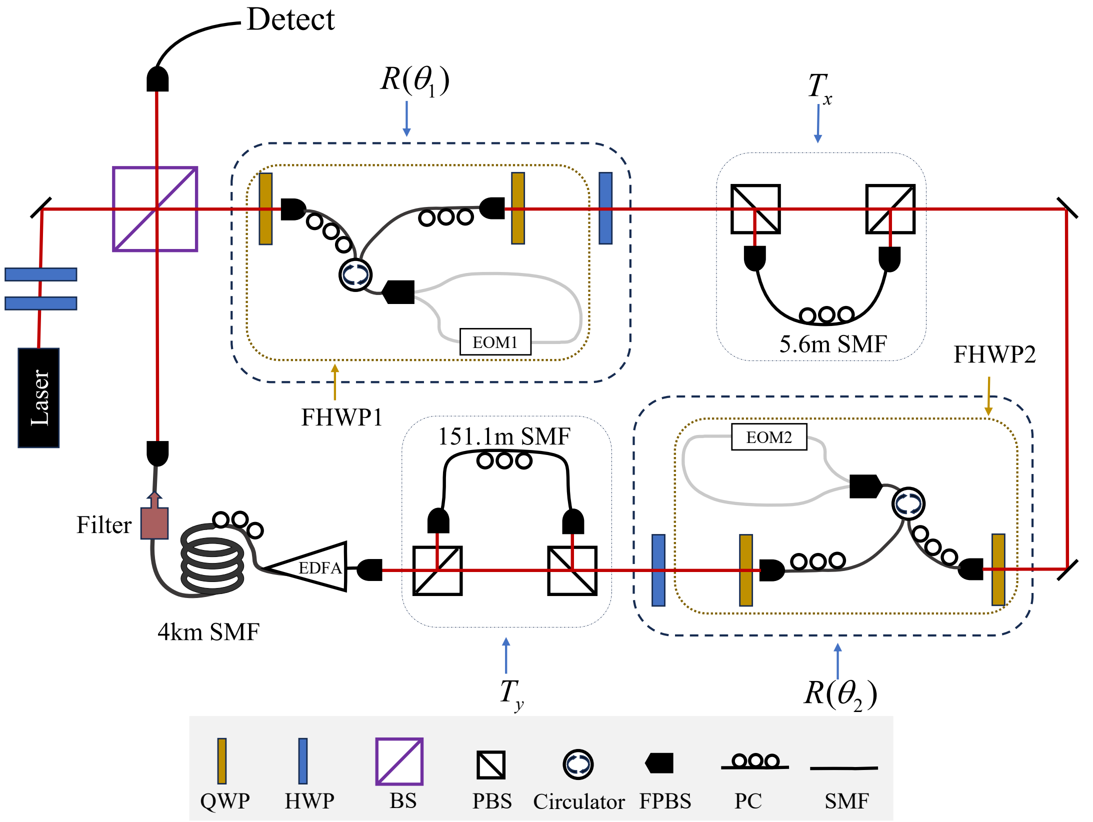

FIG. S4. A detailed experimental schematic. The realization of coin operator $R(\theta)$ and hopping operator $T_x(T_y)$ is shown in detail.

C. Setting for the domain wall

We construct the domain wall by implementing distinct parameter sets $\boldsymbol{\theta}^{\text{in}}$ and $\boldsymbol{\theta}^{\text{out}}$ for the inner and outer regions, respectively. This spatial modulation is achieved through precise control of the electro-optic modulators (EOMs) using a programmed sequence of nanosecond-scale electric pulses, which enable rapid switching between different phase configurations. The resulting discontinuity in $\boldsymbol{\theta}$ values at the boundary creates the desired domain wall structure in the synthetic lattice. The sequences of electric pulses to create the square domain wall and diamond domain wall geometry are sketched in Fig. S5.

D. Initial state preparation for dynamics

In this subsection, we will discuss the settings for initial state's preparation for the dynamical process. First, we introduce the preparation for initial state of dynamic under square domain wall, covering theory and experimental perspectives. Next, we introduce the diamond domain wall case.

For the square domain wall case, we set the initial state localized at the center of the lowest boundary of the domain wall, $(x = 0, y = -3)$, and the lower-right corner of the domain wall $(x = 3, y = -4)$ for the topological phases with 1D boundary states and 0D corner states, respectively. For the strong topological phase with the parameters in Fig. S1b, we see that for the cylinder lattice configurations, i.e., the periodic boundary along y (or x) direction and open boundary along x (or y), the boundary states appear around momentum $k_y = \pm\pi/2$ (or $k_x = \pm\pi/2$). Their spin aligns with the eigenstates of $S_0 = \cos(\frac{\theta_x-\theta_y}{2})\sigma_x + \sin(\frac{\theta_x-\theta_y}{2})\sigma_z$ with eigenvalue $+1$. Thus, we set our initial state as $R^{-1}(\frac{\theta_x^{\text{in}}-\theta_y^{\text{in}}-\pi}{2})|\uparrow\rangle$ in the cases of the rectangular domain wall to observe the dynamics of the chiral edge states.

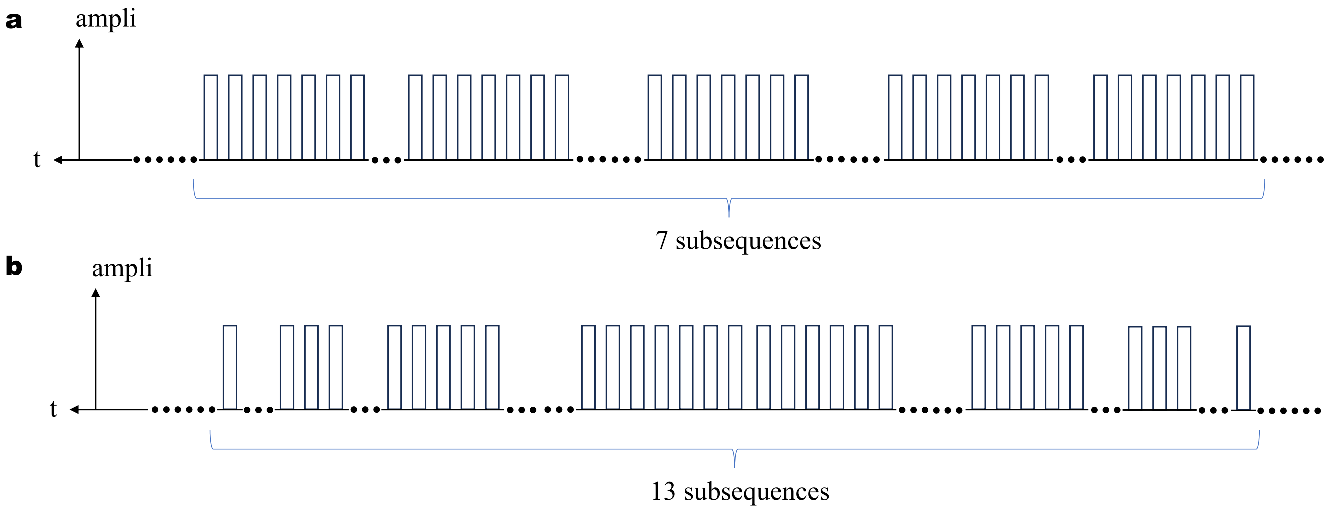

FIG. S5. Sequences of RF signals applied on the EOM for **a** the square domain wall configuration and **b** the diamond domain wall geometry configuration.

For cases with weak topological phases shown in Figs. S1c, d, we choose the internal state of initial state as $R^{-1}(\frac{\theta_x^{\text{in}}-\theta_y^{\text{in}}-\pi}{2})|\uparrow\rangle$. But in these cases, the initial states should overlap with boundary states at subspaces $k_x = \pm\pi/2$ as well as $k_x = 0/\pi$. As a result, the direction of motion in Fig. 4e of the main text and Fig. S9e can not be obtained by their spectra in Figs. S1c and d directly.

For the cases with the 2nd-order topological phases, the corner state emerges due to the nontrivial topological invariants of the subspace k_+ or k_- instead of k_x or k_y subspace, which can be seen from the comparison of Figs. S1e and S2e, i. Thus, we can determine the internal state of the initial state for observing the corner state under square domain wall from the eigenstate analysis of the diamond domain wall cases. The eigenstates under these two types domain walls are shown in Figs. 1d, g of main text, respectively. The lower-right corner states under square domain walls are constructed from the off-diagonal boundary states under diamond domain wall. We focus on zero-energy off-diagonal boundary, which can be from $k_+ = 0$ or $k_+ = \pi$ subspaces, where the topological invariants $2\nu_{\pi/2}^{(0)}\nu_+^{(0)}$ and $-2\nu_0^{(0)}\nu_+^{(0)}$ of inner region are $1/2$ and $-1/2$, respectively, with chiral symmetry operator $S_- = \sin(\frac{\theta_x}{2})\sigma_x - \cos(\frac{\theta_x}{2})\sigma_z$, and parameters in Fig. S1e. The two eigenstates of S_- are $|\phi_\pm\rangle = R^{-1}(\frac{\theta_x+\pi\pm\pi}{2})|\uparrow\rangle$ with eigenvalue ±1. According to the value of topological invariants, the internal state of boundary state at $k_+ = 0$ ($k_+ = \pi$) subspace will be $|\phi_+\rangle$ ($|\phi_-\rangle$). Thus, to manifest the corner state from the dynamics of the pulse with the best performance, we choose the internal state of initial state as $(|\phi_+\rangle \pm |\phi_-\rangle)/\sqrt{2}$ such that the state has the equal overlap with the boundary states at $k_+ = 0$ and $k_+ = \pi$ subspaces. i.e., $R^{-1}(\frac{\theta_x\pm\pi}{2})|\uparrow\rangle$, where the phase difference depends on its position. This position dependency is arisen from the phase difference in the Fourier transformation for $k_+ = 0$ and $k_+ = \pi$ subspaces. Specifically, for the initial states on site ($x = 3, y = -4$) as set in Fig. 4c of main text, the internal state should be $R^{-1}(\frac{\theta_x^{\text{out}}-\pi}{2})|\uparrow\rangle$. And if the initial state is set on the upper inclined side to present one, i.e, site ($x = 4, y = -3$), the internal state should be $R^{-1}(\frac{\theta_x^{\text{out}}+\pi}{2})|\uparrow\rangle$. With this setting, the majority of state will just hop around right-below corner as shown in Fig. 4c of main text.

In the experiment for square domain wall case, we use a polarization beam-splitter (PBS) and two HWPs to initialize the photons at needed, We use the PBS to prepare the photons at horizontal polarization. Due to $R^{-1}(\theta) = R(-\theta)$, the two HWPs initialize the $|\uparrow\rangle$ of the pulse at $R^{-1}(\theta)|\uparrow\rangle$ by setting the parameter $\theta = -4\Delta\phi$ as per Eq. (S72). According to the time sequence of different domain walls shown as Fig. S5, we initialize the pulse at different time bins representing the ($x = 0, y = -3$) and ($x = 3, y = -4$) space set, through making different relative delay of time bins at the EOM, which represents the center of these domain wall sequences and the light pulse creating by the laser diode in Fig. 3 of main text, respectively. For example, to the ($x = 0, y = -3$) and ($x = 3, y = -4$) set, we need to make 2266.53 ns and 3064.01 ns realtive delay, which is the time distance of them.

For these dynamical processes with diamond domain walls, the initial state is put around the middle of right-below domain wall, at $(3,-3)$ and $(4,-3)$. And for the cases with dispersion in Figs. S2f, h and i, the internal state of 0-energy boundary states in $k_+ = 0$ subspace and π-energy boundary states in $k_+ = \pi$ subspace along this domain walls will be $R^{-1}(\frac{\theta_x^{\text{in/out}}+2\pi}{2})|\uparrow\rangle$ because the inner region holds topological invariants $2\nu_{\pi/2}^{(0)}\nu_+^{(0)} = \frac{1}{2}$ with chiral symmetry operator $S_- = \sin(\frac{\theta_x}{2})\sigma_x - \cos(\frac{\theta_x}{2})\sigma_z$. In contrast, it will be $R^{-1}(\frac{\theta_x^{\text{in/out}}+2\pi}{2})|\downarrow\rangle$ for 0-energy boundary states in $k_+ = \pi$ subspace and π-energy boundary states in $k_+ = 0$ subspace with parameters used in Figs. S2h, i, which is orthogonal to former one. Thereby, we can set internal state of initial state as $R^{-1}(\frac{\theta_x^{\text{in/out}}+2\pi}{2})|\uparrow\rangle$ to just overlap with two symmetric branches of boundary states largely, which will hold explicit different

dispersions in A- and B-settings as discussed in Supplementary Note 3 B. Corresponding dynamical processes have been given in Figs. 4d, e, f of main text and Figs. S10a, c, d, where the state will evolve around domain walls and the parity-determined dispersion can be confirmed. In contrast, there is no boundary around domain walls along right-below domain wall direction for the case in Fig. S2g. As a reflection, the state will scatter into the bulk rapidly, shown in Figs. S9f and S10b.

To implement initial states for diamond domain walls we use the same setup to initialize the polarization and time-bin position of photon. We just need to set different rotation angles of two HWPs and delay for the center of domain wall sequence, according to the parameter inside or outside domain wall and above space sets in diamond domain wall.

Supplementary Note 5. QUANTIFICATION DETAILS

A. Definition of IPR and center-of-mass displacement

In this section, we present specific definitions of some physical quantities, which are used in the main text to distinguish different topological phases. Specifically, the inverse participation ratio (IPR) defined as

$$\mathrm{IPR} = \sum_{\boldsymbol{r}} (|\langle \boldsymbol{r}, \uparrow |\psi_n\rangle|^2 + |\langle \boldsymbol{r}, \downarrow |\psi_n\rangle|^2)^2, \tag{S73}$$

characterizes the pulse's localization degree, with values ranging from $1/N$ (fully extended state) to 1 (perfectly localized state), where a larger IPR indicates stronger spatial confinement and N is the number of sites.

We define the center-of-mass displacement d_c as:

$$d_c = \frac{\langle \bar{\psi}_n | x_c | \bar{\psi}_n \rangle}{\langle \bar{\psi}_n | \bar{\psi}_n \rangle}, \quad |\bar{\psi}_n\rangle = P_b |\psi_n\rangle, \tag{S74}$$

where P_b projects onto sites near domain walls, and x_c is encoded as Fig. S6a (Fig. S7a when the pulse's distribution doesn't pass through half the domain wall, or as Fig. S6b (Fig. S7b) goes through half the domain wall.

Using these two forms of distance can further illustrate the distinction that unchiral boundary states allow bidirectional propagation, whereas chiral boundary states permit only unidirectional propagation.

The practical computation is shown in Figs. S6 and S7. For the square domain wall case, we define the center-of-mass displacement of the sets near the domain wall as Fig. S6a. And Fig. S6b shows the longer distance propagation, when the pulse's distribution spreads through the half the length of square domain wall. For the corner initial state of 2nd-order topological case under square domain wall, we just need to shift the $x_c = 0$ to the right-below corner of square domain wall. Considering the parity of sets, we define the center-of-mass displacement of the sets near the diamond domain wall as Fig.S7a. And Fig.S7b depicts the longer distance propagation, when the pulse's distribution spreads through half the diamond domain wall.

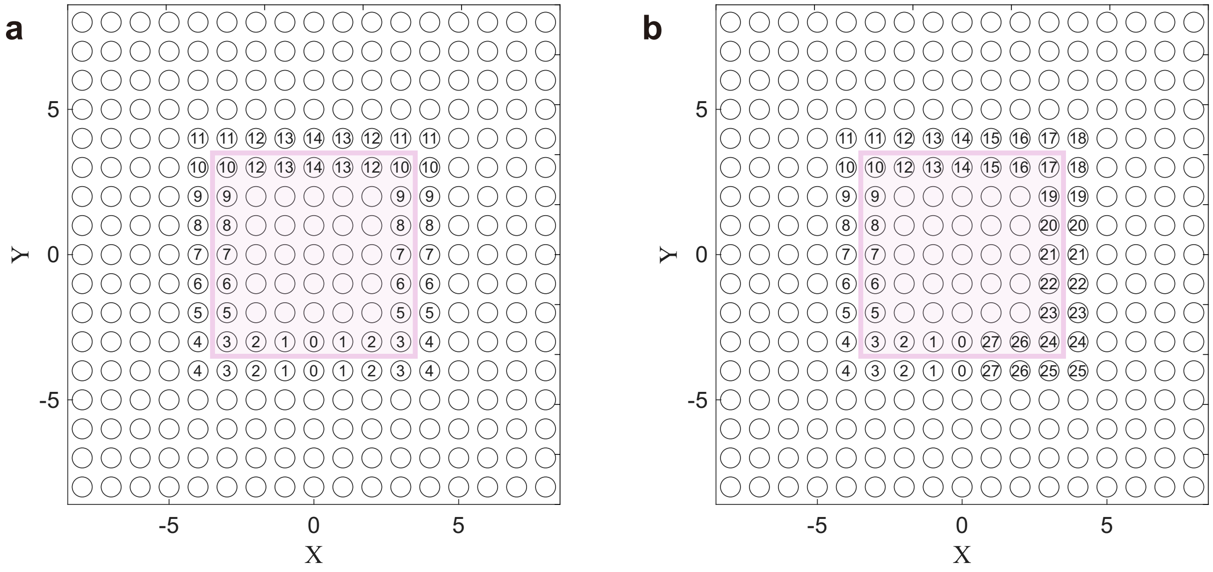

FIG. S6. The sketch of sequence for center-of-mass displacement under square domain wall. **a** for the pulse's distribution just dominates at two adjacent parts of square domain wall. **b** for the pulse's distribution pass through half the square domain wall.

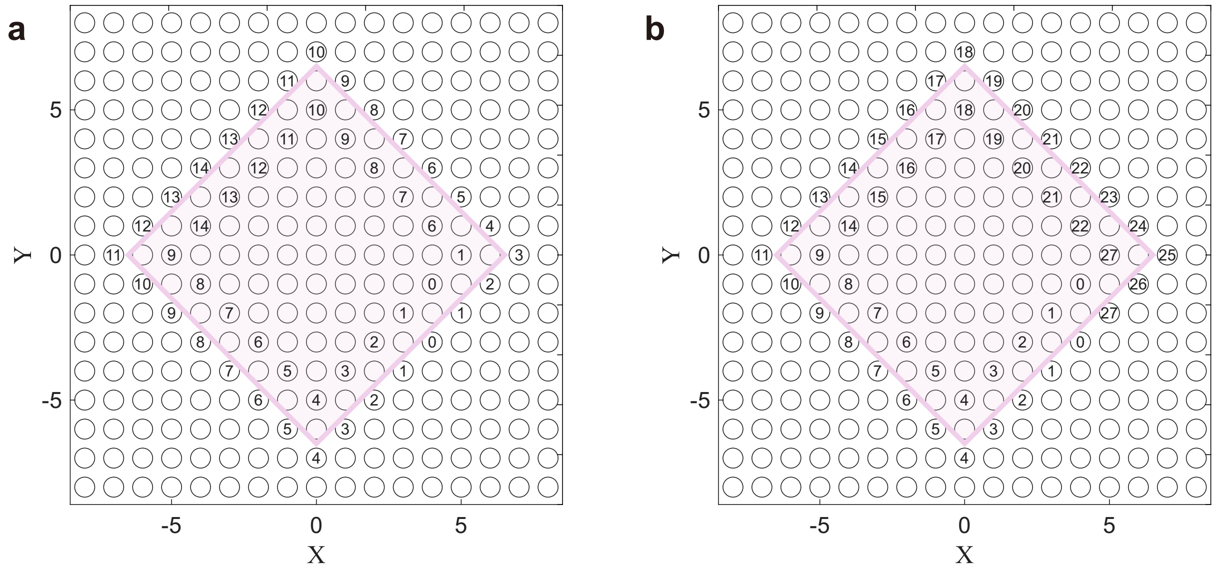

FIG. S7. The sketch of sequence for center-of-mass displacement under diamond domain wall. **a** for the pulse's distribution just dominates at two adjacent parts of diamond domain wall. **b** for the pulse's distribution pass through half the diamond domain wall.

B. IPR and center-of-mass displacement for enlarged system sizes

We theoretically simulate the distinct scaling behaviors for different topological regimes. For the square domain-wall geometry [Fig. S8(a)], the center-of-mass displacement exhibits a linear dependence on the evolution time that is robust with respect to the domain-wall size. Consistent with the results presented in the main text, the chiral, weak, and second-order boundary states exhibit linear growth with a large slope, linear growth with a small but finite slope, and bounded oscillations without growth, respectively.

For the diamond domain-wall geometry [Fig. S8(c)], the center-of-mass dynamics in the strong topological phase progressively converge to a clear linear relation as the domain-wall size increases, providing more evident signatures of the underlying transport behavior. In contrast, weak boundary states consistently exhibit linear growth with a small but finite slope. Notably, the two types of weak boundary states show different growth rates due to their distinct dispersion relations, which determine different group velocities.

The scaling of the IPR further distinguishes strong, weak, and second-order topological boundary states from a complementary perspective. For the square domain-wall geometry [Fig. S8(b)], the corner states remain strongly localized, with the IPR approaching a stable value of approximately 0.6, independent of the system size, whereas the IPR for strong and weak boundary states decreases over time, reflecting their delocalized nature (albeit with different decay rates). The size-independent saturation of the IPR for second-order topological boundary states is consistent with the real-space dynamics: the evolved state exhibits nearly steady oscillations around two sets of sites near the lower-right corner of the square domain wall. In the diamond domain-wall geometry [Fig. S8(d)], the IPRs of both strong and weak boundary states decrease over time without a clear distinction, consistent with their extended boundary character in this geometry.

These numerical results provide a qualitative characterization of the scaling behavior and help clarify the distinction between strong, weak, and second-order boundary states, thereby complementing the experimental observations where finite system size limits the visibility of these differences.

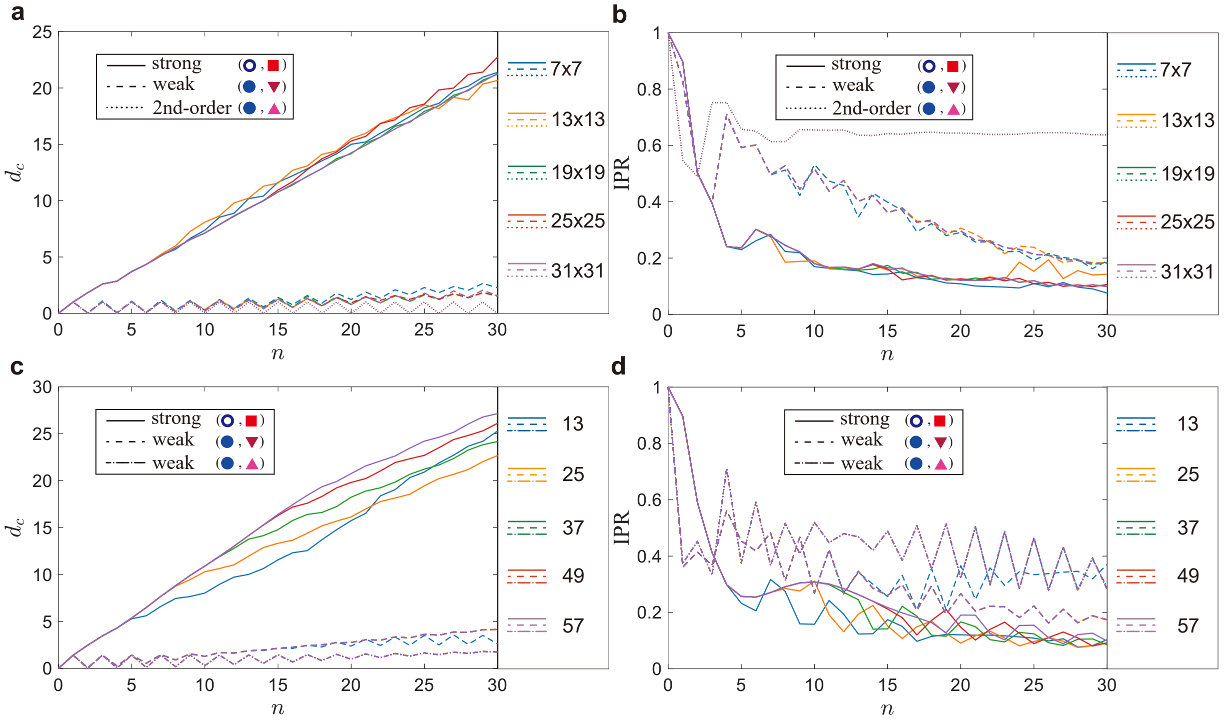

FIG. S8. The center-of-mass position (d_c) and inverse participation ratio (IPR) are plotted as functions of the time step n. (a,b) and (c,d) correspond to the square and diamond domain wall configurations, respectively, where the numbers represent the size of square domain wall and axis length of diamond domain wall respectively.

Supplementary Note 6. OTHER RESULTS OF EXPERIMENT

In this section, we provide the dynamics in another weak topological phase in Supplementary Note 6 A, and the inverse propagation process for even setting ($A-$setting) under diamond domain wall in Supplementary Note 6 B.

A. Another topological case

In this subsection, we will discuss the other dynamics for weak topological phases with unchiral boundary states. For this weak topological phase, the parameters of inner region and outer region is $\boldsymbol{\theta}^{\text{in}} = (0.6\pi, 0)$ and $\boldsymbol{\theta}^{\text{out}} = (-0.6\pi, 0.2\pi)$, as the • and ⋆ in Fig. S1a phase diagram, respectively. Under the square domain wall, the system has the same unchiral edge state, as shown in Figs. S9a, b, analogous to the weak topological case in Fig. 2b of main text. However, under the diamond domain wall, the system has the off-diagonal unchiral edge state in this topological case as Fig. S9c, contrasting with the weak topological phase hosting diagonal unchiral edge state in the Fig. 2e of main text. Therefore, when we initialize the pulse at right-below domain wall, at the site $(x = 3, y = -3)$ with spin $R^{-1}(\frac{\theta_x^{\text{out}}+2\pi}{2})|\uparrow\rangle$, the pulse will propagate into the bulk, as Fig. S9f.

B. Inverse propagation due to the setting's parity

In this subsection, we will discuss inverse propagation induced by the different parity of sets. Regardless of the odd and even values of $x + y$ of initial sites(corresponding to B-setting and A-setting) under diamond domain wall in Fig. 3(b1) and Fig. S10(a), the pulse always propagates along a certain direction of the domain wall, indicating the chirality of edge state in strong topological phase. Since the pulse is not initialized at the edge state position, the pulse still moves to the bulk, in Fig. S10(b). However, the pulse does not always move along the same direction for A and B setting in weak topology. The pulse may propagate to inverse direction when the pulse is initialized at site with even value of $x + y$ (A-setting) in Figs. S10(c,d), comparing to the dynamics when the pulse is initialized at at site with odd value of $x + y$ (B-setting) in Figs. 3(b2, b3).

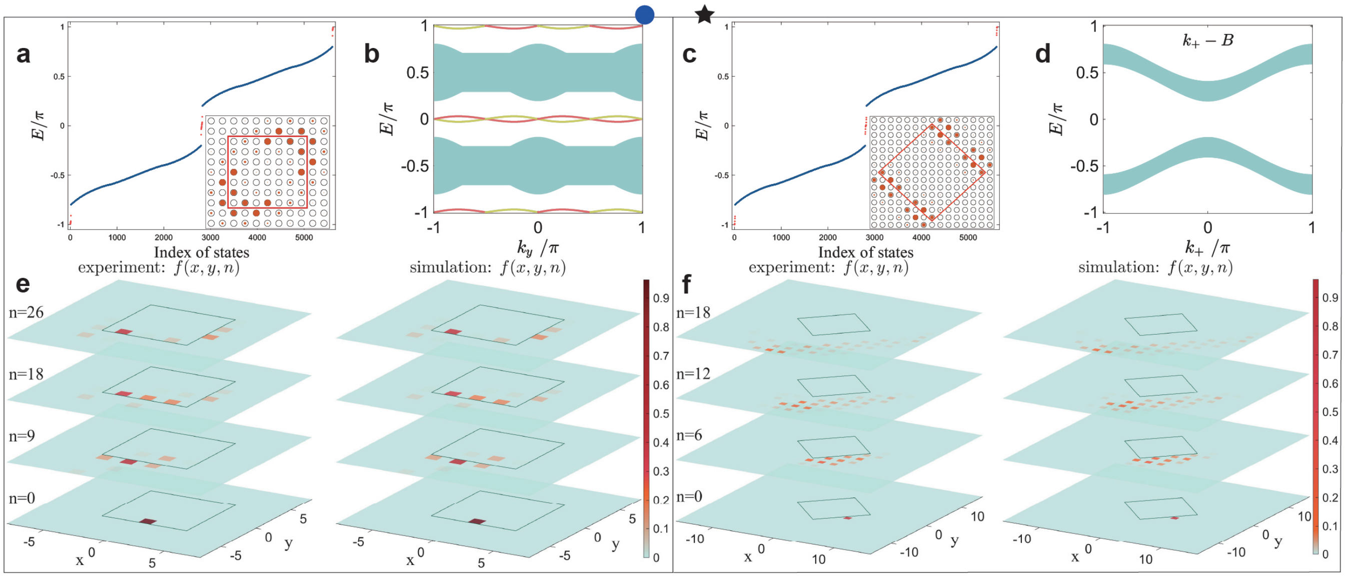

FIG. S9. The spectrum and dynamics for another weak topological case in square and diamond domain walls, where $\boldsymbol{\theta}^{\text{in}}$ and $\boldsymbol{\theta}^{\text{out}}$ are denoted by • and ⋆, respectively. **a**, **c** Energy spectrum with the enclosed domain wall and typical edge state distribution. **b**, **d** Corresponding dispersion relations for square and diamond domain wall. **e**, **f** Experimental and theoretical simulated dynamics under square and diamond domain wall, showing the pulse's 2D spatial distribution for $n = 0,\ 9,\ 19,\ 26$ in **e** and $n = 0,\ 6,\ 12,\ 18$ in **f**.

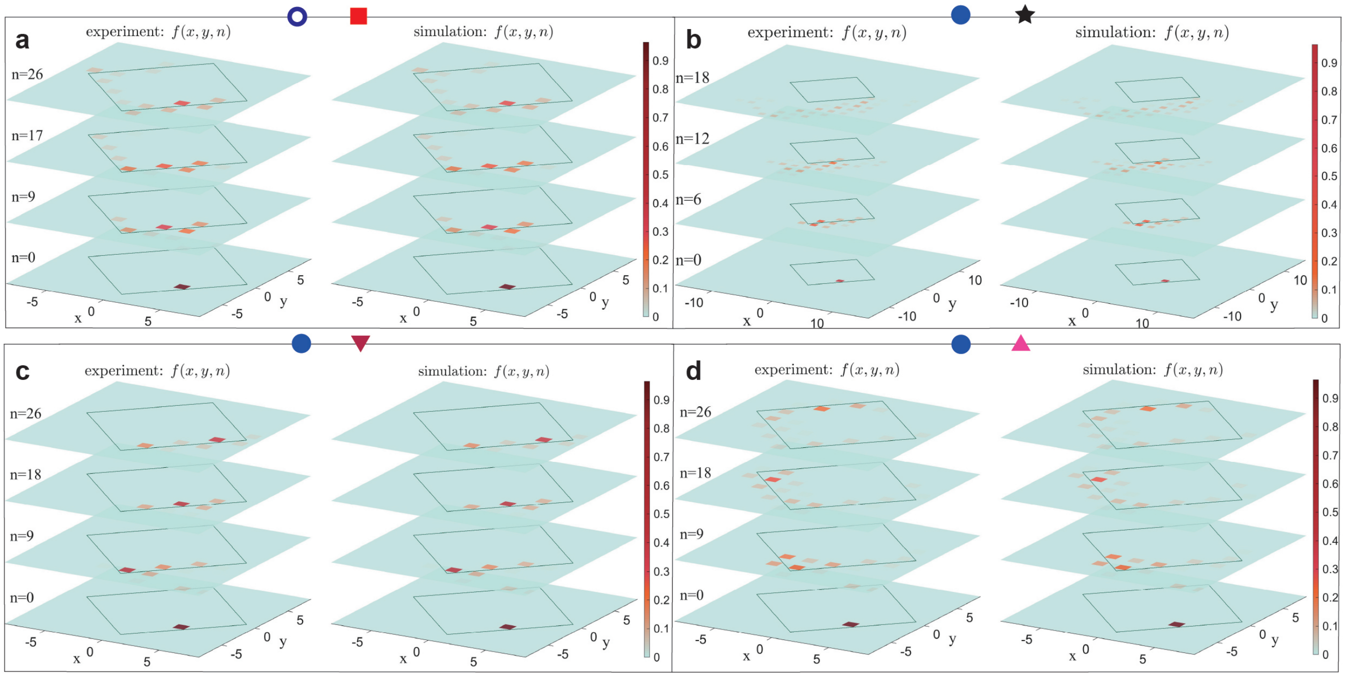

FIG. S10. The dynamics when the pulse is initialized in even setting (A-setting). The symbols ○ and • denote $\boldsymbol{\theta}^{\text{in}}$, which are $(\theta_x, \theta_y) = (1.3\pi, 0)$ and $(0.6\pi, 0)$; ■, ⋆, ▼, and ▲ denote $\boldsymbol{\theta}^{\text{out}}$, which are $(\theta_x, \theta_y) = (0.3\pi, 1.2\pi),\ (-0.6\pi, 0.2\pi),\ (1.1\pi, 1.6\pi)$ and $(-1.042\pi, 1.6\pi)$, respectively. For each case, we provide the experimental and simulated dynamics, showing the pulse's 2D probability distribution for $n = 0,\ 9,\ 18,\ 26$ in (a,c,d) and $n = 0,\ 6,\ 12,\ 18$ in (b). **a** the pulse propagates clockwise for even setting in the strong topological case. **b** the pulse still moves into the bulk for even setting in another weak topological case. **c** the pulse propagates anti-clockwise at the early steps in even setting in the weak topological case. **d** the pulse propagates anti-clockwise for even setting in the weak topological case, which is the 2nd-order topological case.

[1] D. Xiao, M.-C. Chang, and Q. Niu, Berry phase effects on electronic properties, Rev. Mod. Phys. **82**, 1959 (2010).

[2] J. Ahn, S. Park, D. Kim, Y. Kim, and B.-J. Yang, Stiefel-whitney classes and topological phases in band theory, Chin. Phys. B **28**, 117101 (2019).

[3] C. Chen, X. Ding, J. Qin, Y. He, Y.-H. Luo, M.-C. Chen, C. Liu, X.-L. Wang, W.-J. Zhang, H. Li, L.-X. You, Z. Wang, D.-W. Wang, B. C. Sanders, C.-Y. Lu, and J.-W. Pan, Observation of topologically protected edge states in a photonic two-dimensional quantum walk, Phys. Rev. Lett. **121**, 100502 (2018).

[4] C. Chen, X. Ding, J. Qin, J. Wu, Y. He, C.-Y. Lu, L. Li, X.-J. Liu, B. C. Sanders, and J.-W. Pan, Topological spin texture of chiral edge states in photonic two-dimensional quantum walks, Phys. Rev. Lett. **129**, 046401 (2022).

[5] M. S. Rudner, N. H. Lindner, E. Berg, and M. Levin, Anomalous edge states and the bulk-edge correspondence for periodically driven two-dimensional systems, Phys. Rev. X **3**, 031005 (2013).

[6] S. Ryu, A. P. Schnyder, A. Furusaki, and A. W. W. Ludwig, Topological insulators and superconductors: tenfold way and dimensional hierarchy, New J. Phys **12**, 065010 (2010).

[7] C.-K. Chiu, J. C. Y. Teo, A. P. Schnyder, and S. Ryu, Classification of topological quantum matter with symmetries, Rev. Mod. Phys. **88**, 035005 (2016).

[8] J. K. Asbóth and H. Obuse, Bulk-boundary correspondence for chiral symmetric quantum walks, Phys. Rev. B **88**, 121406 (2013).
